# Non-contact neuromodulation of the human autonomic nervous system function *via* different odors: Sex, menstrual cycle, and odor dose- and duration-specific effects

**DOI:** 10.3389/fnins.2022.950282

**Published:** 2022-10-12

**Authors:** Ashim Maharjan, Prashanna Khwaounjoo, Mei Peng, Yusuf Ozgur Cakmak

**Affiliations:** ^1^Department of Anatomy, School of Biomedical Sciences, University of Otago, Dunedin, New Zealand; ^2^Medical Technologies Centre of Research Excellence, Auckland, New Zealand; ^3^Auckland Bioengineering Institute, University of Auckland, Auckland, New Zealand; ^4^Sensory Neuroscience Laboratory, Department of Food Science, University of Otago, Dunedin, New Zealand; ^5^Brain Health Research Centre, Dunedin, New Zealand; ^6^Centre for Bioengineering and Nanotechnology, Point of Care Technologies, University of Otago, Dunedin, New Zealand

**Keywords:** non-contact neuromodulation, autonomic nervous system (ANS), odor stimulation, parasympathetic nervous system (PNS), odor concentration, electrocardiogram (ECG), vagus, HRV (heart-rate variability)

## Abstract

In recent decades, it has been uncovered that the autonomic nervous system (ANS) can be influenced using non-contact neuromodulation *via* odor stimulation. Increasing parasympathetic-vagal activation of the ANS is integral to improving the sympathovagal balance between the sympathetic- and parasympathetic nervous systems, which is often imbalanced in several chronic inflammatory disorders, such as rheumatoid arthritis and inflammatory bowel diseases. Although research into olfactory stimulation has been observed on the ANS, it is still lacking in the exploration of odor concentration and odor-specific effects. This is particularly the case as research has not utilized specified tools, such as the olfactometer to provide precise odor delivery. Furthermore, no research has compared the results in separate sex cohorts to investigate the role of sex or the menstrual stage on the subsequent interactions. In this study, we investigated the olfactory stimulation effects of four natural odors (mushroom, lavender, jasmine, and rose) in three concentrations (low, moderate, and high) on the ANS. To observe activity from the ANS, we used an electrocardiogram (ECG) based heart rate variability (HRV) and eye-tracker technology (pupil diameter). We found for the first time in literature that there were acute dose- and duration-specific odor effects of odors on the ANS. We also found sex and menstrual cycle effects in this interaction. Furthermore, there were stark distinctions in sympathovagal activity dependent ANS activation (HRV) in comparison to the oculomotor nerve-parasympathetic/cervical sympathetic nerves dependent ANS responses (pupil diameter). Sympathovagal activity dependent HRV showed odor, sex, and menstrual-stage interactions in both divisions of the ANS while the pupil responses only indicated increased sympathetic activation. These results shed light on the use of odor-specific stimulation to modulate the ANS activity in the context of sex and the menstrual stage. Future studies should be performed using a chronic odor delivery design to investigate the long-term effects of odors on the ANS.

**Clinical trial registration:**

Australian New Zealand Clinical Trials Registry, identifier [ACTRN12622000415707].

## Introduction

The field of neuromodulation covers a broad spectrum of topics but, overall, pertains to the use of a stimulus on specific parts of the body or brain. Neuromodulation can be performed in an invasive or non-invasive manner. Invasive neuromodulation, for example, can be through the use of invasive deep brain stimulation where implant electrodes are placed in specific regions of the brain (Yuan and Silberstein, 2016). Non-invasive neuromodulation, for instance, can be performed using manual acupuncture techniques (Huang et al., 2005; Li et al., 2005; Tokumaru and Chen, 2005; Chang et al., 2008) or, more commonly in current literature, the use of transcutaneous electrical nerve stimulation over specific areas of the body (Yuan and Silberstein, 2016; Maharjan et al., 2018, 2019, 2021). More recently, there has also been a shift of focus toward using non-contact neuromodulation. Moving neuromodulation toward non-invasive methods aids in a more simplified set-up, reduces associated risks with invasive maneuvers and allows the treatment process to be performed as a form of self-treatment by the patient, forgoing the need for skilled technicians or clinicians in the treatment process. One particular form of this method is through the use of odor-olfactory stimulation. In the existing literature, the use of olfactory stimulation using odors is vast, ranging from its use for numerous physiological and psychological states, such as sleep (Lewith et al., 2005; Wheatley, 2005; Chien et al., 2012), anxiety (Buchbauer and Jirovetz, 1994; Buchbauer, 1996), and attention (Seo et al., 2010). Due to its health benefits, olfactory stimulation is also used to treat conditions, such as depression or anxiety disorders (Buchbauer and Jirovetz, 1994; Buchbauer, 1996). In recent decades, there has been a shift in olfactory research to modulate the parasympathetic-vagal tone of the autonomic nervous system (ANS).

The ANS is an integral part of the peripheral nervous system and is responsible for the regulation of involuntary physiological-homeostatic processes, such as heart rate (HR), blood pressure (BP), respiration, digestion, pupillary contraction of the eyes, thermoregulation, and even sexual arousal (Waxenbaum et al., 2021). There are two different divisions of the ANS: the sympathetic- and parasympathetic nervous systems (SNS and PNS, respectively) (Paxinos, 1990; Waxenbaum et al., 2021). SNS and the PNS contain both the afferent and efferent fibers that provide sensory input and motor output, in communication with both the central nervous system and other parts of the peripheral nervous system. It is well understood that activation of the SNS leads to overall elevated activity and attention, termed the “fight or flight” response. Here, BP and HR increase while other functions, such as glycogenolysis, ensue, and gastrointestinal peristalsis ceases (Koopman et al., 2011). In comparison, the PNS promotes the well-known “rest and digest” phenomenon, where HR and BP decrease while gastrointestinal peristalsis and digestion are engaged (Koopman et al., 2011; Kenney and Ganta, 2014). The balance between the activation of the SNS and PNS is referred to as sympathovagal balance, which was popularized by the attention drawn to the field of ANS research (Eckberg, 1997). Discrepancies in sympathovagal balance due to either the over-activity of the SNS or reduced vagal activity (PNS) are associated with increased mortality or impaired immune response in numerous chronic inflammatory disorders (Electrophysiology, 1996; Bonaz et al., 2016; Kalliolias and Ivashkiv, 2016; Koopman et al., 2016; Yap et al., 2020). An increase in vagal-parasympathetic activation is associated with increased protection against systemic inflammation *via* the regulation of the cytokine release from the spleen through the activation of the inflammatory reflex (Borovikova et al., 2000; Tracey, 2002; Chavan et al., 2017).

There are numerous methods to measure the activity of the ANS; however, the most common is the use of electrocardiogram (ECG), which can be used to measure heart-rate variability (HRV) parameters (Electrophysiology, 1996; Shaffer and Ginsberg, 2017). HRV represents oscillation of the heart, which, when healthy, is complex, non-linear, and flexible in order to cope with a rapidly changing environment (Shaffer and Ginsberg, 2017). ECGs measure the electrical activity of the heart and can be used to extract HRV parameters from two distinct domains: time and frequency. Time-domain ECG analyses focus on the heart rate at any point in time or the intervals between successive normal complexes. Measures used in this domain focus on the ‘QRS’ complex and the normal-to-normal intervals between adjacent QRS complexes (Electrophysiology, 1996). Frequency-domain analyses focus on the power spectral density, which provides information on how power (variance in some cases) is distributed as a function of frequency. An alternative method to measure ANS activation is through pupillary dilation from an eye-tracker (Finke et al., 2017; Gajardo et al., 2019). Although previously understood to be primarily influenced by a change in luminance of the visual environment, heightened sensory responses, such as that from the ANS, can play a significant effect on pupillary changes (Larsen and Waters, 2018). It is well understood that activity of the pupils correlates with HR and skin conductance, both measures of the ANS (Bradley et al., 2008). Control of pupil constriction occurs through the parasympathetic fibers of the oculomotor nerve while cervical sympathetic nerves control pupil dilation (Ruskell, 2003; Jodoin et al., 2015; Mcdougal and Gamlin, 2015). It is currently unclear whether pupil activity is connected to the vagus network as vagus nerve stimulation has shown inconsistent modulation of ANS when measuring autonomic activity from the pupils (Jodoin et al., 2015; Schevernels et al., 2016; Warren et al., 2018; Keute et al., 2019).

Olfactory stimulation was initially performed under a branch of an ancient traditional herbal (‘aromatherapeutic’) medicine thousands of years ago (Cooke and Ernst, 2000; Huang and Capdevila, 2017). Research using olfactory stimulation has shown effectiveness in modulating both branches of the ANS (Alaoui-Ismaili et al., 1997; Ikei et al., 2015; Kroupi et al., 2016; Nomura et al., 2016; Huang and Capdevila, 2017, Tonacci et al., 2019). The use of certain odors, such as lavender or jasmine, as a form of olfactory stimulation, is understood to be able to modulate the sympathovagal balance (Kuroda et al., 2005; Igarashi, 2013; Tonacci et al., 2019). Primarily observed in the frequency-domain ECG, odors modulated parasympathetic, high-frequency (HF) power (Kuroda et al., 2005; Ikei et al., 2015; Nomura et al., 2016; Tonacci et al., 2019) and the ratio of low-high frequency (the LF/HF ratio) of the ANS (Tonacci et al., 2019). However, there is limited research on olfactory stimulation on the ANS using an ECG measuring HRV parameters in the time domain. A measure of the time-domain ECG would allow the distinction of ANS effects irrespective of the breathing rate (Electrophysiology, 1996; Shaffer and Ginsberg, 2017). Furthermore, little research has used more than one technique to monitor the activities of ANS. Joint applications of ECG and the eye-tracker (*via* pupillary dilation measures) would respectively allow the measure of vagus-dependent and oculomotor/cervical sympathetic nerves-dependent ANS activations.

A variable that is often ignored in the field of olfactory research on the ANS is the role of sex or the menstrual stage (in female participants). It is well understood that sex can play a large role in olfactory perception (Toulouse and Vaschide, 1899a,b; Wysocki et al., 1989), olfactory performance (Toulouse and Vaschide, 1899a,b; Doty et al., 1984, 1985, 1997; Engen, 1987; Wysocki et al., 1989; Klukty, 1990), and even the anatomy of sex-specific olfactory processes (Shaywitz et al., 1995; Gilbert et al., 1997; Henkins, 1997; Levy et al., 1997; Hornung et al., 1999). Furthermore, there are also reported sex differences in the activation patterns of the ANS (Ryan et al., 1994; Liao et al., 1995; Madden and Savard, 1995; Yamasaki et al., 1996; Dart et al., 2002; Shaffer and Ginsberg, 2017). There are also evident differences in olfactory performances dependent on the menstrual stage of the female participant (Le Magnen, 1952; Schneider and Wolf, 1955; Vierling and Rock, 1967; Koster, 1968; Good et al., 1976; Mair et al., 1978; Doty et al., 1981; Henkins, 1997; Moriyama and Kurahashi, 2000; Osterlund and Hurd, 2001; Sundermann et al., 2006; Derntl et al., 2013). This also extends to differences in ANS activation due to sex or the menstrual stage (Madden and Savard, 1995; Minson et al., 2000a,b; Dart et al., 2002). Of the existing literature on the effect of olfactory stimulation on the ANS, there are only two articles that have not used a mixed cohort (Ikei et al., 2015; Nomura et al., 2016). However, neither investigated the effect of sex on ANS parameters.

Furthermore, with olfactory stimulation in the existing literature, most researchers have used a vast range of techniques to deliver odors. A popular method is to use small lidded-glass bottles, thanks to their cost and accessibility. With this method, odors are stored in a small glass bottle before use (Kroupi et al., 2016; Maharjan et al., 2018, 2019). However, there are several limitations to such a procedure. This technique cannot guarantee the rate of odor concentration over exposure time, control the temperature of odor delivery, and the measurement of the parts-per-million (PPM) delivered to the nostril (odor delivery using this method is usually outside the nasal passage, ∼ 2–3 cm away). More recently, research has made some attempts to optimize the practice of odor delivery to combat the aforementioned limitations using techniques, such as sniffin’ sticks (Doty et al., 1985, 1995; Tonacci et al., 2019), diffusers (Huang and Capdevila, 2017), odor bags with the delivery tube in the nose (Ikei et al., 2015) or even airtight bottles that have an air pump system (Kuroda et al., 2005). However, these techniques still fail to combat the control of the odor flow rate (keeping constant strength of the odor delivered). One technique that allows for such control in odor delivery is the use of the olfactometer. This device allows for the creation of well-defined and reproducible smell stimuli in the nose without tactile or thermal stimulation while creating a clean airflow at the nose outlet in which stimuli of different types, concentrations, and durations can be virtually embedded at any desired time by the experimenter’s settings. It can also control for the change in the flow rate from control (odorless airflow) and odor delivery, keeping the temperature and humidity of odor delivery constant. Therefore, using this novel technology, the aim of this article was to observe the neuromodulatory effects of olfactory stimulation *via* the olfactometer on the ANS measured through ECG and the eye-tracker. This was explored in both sex cohorts, further distinguishing between follicular and luteal menstrual stages in the female participants.

## Methods

### Participants

There were 21 healthy-adult, right-handed male (age range of 20–33 years, mean = 26.57 years, st.d. = ± 3.01 years) and 21 healthy-adult, right handed female (age range of 19–33 years, mean = 27.23 years, st.d. = ± 3.58 years) participants recruited for this study. Our age range was kept to a minimum to avoid the effects of aging on HRV parameters indicated in previous literature (Pfeifer et al., 1983; Yamasaki et al., 1996; Shaffer and Ginsberg, 2017). Specifically, we kept the age range within 2 decades to minimize the effect of aging on HRV parameters. The participants also performed experiments in the same time frames (either morning, 8–9 AM, 9–11 AM, or afternoons, 1–3 PM, 3–4 PM) to avoid the effect of time of day present in HRV research (Yamasaki et al., 1996; Shaffer and Ginsberg, 2017). Female participants—experiments were performed in two stages of the menstrual cycle (follicular and luteal stages). Prior to each session, the participants filled out a self-reported information sheet that indicated they had no health impairments, no visual or olfactory loss, had no history of smoking, and had a BMI value of <30. None of the participants consumed food or caffeine <2 h and alcohol <48 h of the experiment. All the female participants were off hormonal contraceptives, with no history of menopause, pregnancy or breastfeeding, and did not report experiencing irregular menstrual cycles during and at least 3 months before the study. These factors were included in the inclusion criteria as they have been shown to influence olfactory performances, in addition to creating difficulty in estimating regular menstrual cycles (Woodward et al., 2015). Female volunteers were also asked to report the 1st day of their most recent menstrual cycle. This allowed calculations of the follicular and luteal stages of their menstrual cycle. We used Days 1–14 of the menstrual cycle as the estimated follicular days and Days 18–28 of the menstrual cycle as the estimated luteal days for the study. This is in line with previous research, which has conducted the estimates of the menstrual cycle in the same manner (Derntl et al., 2013). All the participants gave informed, written consent to participate in the experiment in accordance with the Declaration of Helsinki and met the inclusion criteria set for the experiment. This study was approved by the Otago Human Participants Ethics Committee (reference: H20/123) and the Australian New Zealand Clinical Trials Registry (ANZCTR No.: 12622000415707).

### Visual analog scale

The Visual analog scale (VAS) is an effective retest measure in human studies for a variety of psychosomatic information and subjective ratings, including stimulus intensity and hedonic value of stimuli (Thompson and Campbell, 1977; Hill and Blundell, 1982, 1990; De Castro and Elmore, 1988; Mattes, 1990; Westrate, 1992; De Graaf, 1993; Flint et al., 2000; Stubbs et al., 2000). In this study, the VAS tests were used to measure subjective ratings across two different parameters. This included odor intensity and hedonic value of odor. For odor intensity, the VAS was presented on a horizontal scale from 0 (not at all) to 100 (very much). For the hedonic value of odor, the VAS was presented on a horizontal scale from −100 (unpleasant) to 100 (pleasant). We measured odor intensity and hedonic value of odor to ensure that the participants could detect odor perception (intensity and hedonic value) at all three concentrations (low, moderate, and high) in comparison to sham stimulation. The VAS tests were made using the software Qualtrics (SAP 2021, Provo-Utah, United States).

### Electrocardiogram

An ECG device (eMotions FAROS 360, SN:1724489, Mega Electronics Ltd., Pioneerinkatu 6, FI-70800 Kuopio, Finland) (sampling frequency, 1,000 Hz) was used to acquire HRV metrics from two domains of the ECG, time, and frequency. The time and frequency HRV metrics were extracted using the KUBIOS Premium HRV analysis software (v.3.3.0, HRV analysis, Kubios Oy, Finland). For the time domain, we utilized the root mean square of successive differences between normal heartbeats (RMSSD) and stress index (SI), which represents activation from the PNS and SNS, respectively. RMSSD reflects the beat-to-beat variance in HR and is considered the primary measure in time domain ECG analysis, used to estimate vagal tone changes reflected in HRV (Electrophysiology, 1996; Shaffer and Ginsberg, 2017). In contrast, SI measures the activity from the SNS, calculated by the square root of the well-known Baevsky’s stress index model [*see* (Baevsky and Chernikova, 2017) *for a review on this model*]. For the frequency-domain ECG, we extracted the low-frequency (LF) power, high-frequency (HF) power, and the LF/HF ratio. It is evident in the literature that the HF power component is reflective of efferent vagal activity, observed in studies of electrical vagal stimulation, muscarinic receptor blockade, and vagotomy (Akselrod et al., 1981; Malliani et al., 1991). However, for the LF power component, there is some controversy regarding whether it represents sympathetic modulation (Rimoldi et al., 1990; Kamath and Fallen, 1993) or both sympathetic and vagal influences (Akselrod et al., 1981). Finally, the LF/HF ratio is a commonly used parameter to observe sympathovagal balance (Electrophysiology, 1996). At the start of the experiment, the participant was seated, and the ECG was placed on the skin below the sternum. The ECG was secured with a band encircling the torso, allowing for easy adjustment to ensure a fixed position. It was removed at the end of the post-stimulation period.

### Olfactometer

**FIGURE 1:**
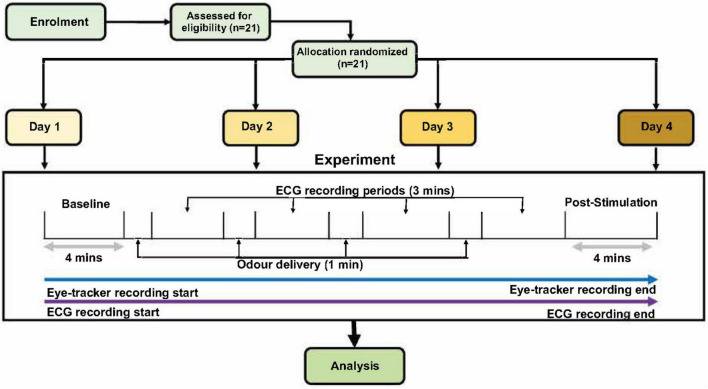
The schematic for the experimental procedures. After 4 min of baseline recording, each participant received four different odor stimuli (low, moderate, and high concentration of one odor, and one sham stimulation with distilled water). Only one odor was present per day; therefore, the three concentrations present on each day were of the same odor (either lavender, jasmine, mushroom or rose) but in three concentrations and one sham. Order of days and odor stimuli were randomized across all the participants. Odor presentation was for 1 min per odor delivery, with 3-min inter-stimulus wash-out periods. Finally, there were a 4-min post-stimulation period to conclude each of the experimental days. The only difference in experiment design for the male and female cohorts was that, in the female cohort, this experiment was repeated two times in two stages of the menstrual cycle (follicular and luteal).

The odors for the experiment were delivered using an olfactometer (Modular Olfactometer Ol023 Medical Device, Burghart Messtechnik GmbH, Germany). There were a total of four different odors chosen for this study. *Lavender* [New Zealand ‘True Lavender’; Lavandula Angustifolia, components—linalool (13%), linalyl acetate (48%), octan-3-one (1.9%), 1–8 Cineole (0.4%), and camphor (0.25%)] and *jasmine* [Jasmine Absolute; Jasminum Officinale, natural plant extract (flowers) 100% pure therapeutic grade essential oil, components—benzyl acetate (24.7%), benzyl benzoate (12.2%), Phytol (11.7%), Linalool (7.4%), Benzyl alcohol (3.3%), Eugenol (2.5%), Cresols (2%), Nerolidol (0.7), and Methyl benzoate (0.2%)] were chosen due to their properties of exhibiting activation in both divisions of the ANS (Dayawansa et al., 2003; Kuroda et al., 2005; Ikei et al., 2015). *Rose* [Rose Anatolia: cosmetic grade, natural plant extract (flowers), components: Citronellol (25%), Alcohols—C11-14- iso-, C13-rich (25%), hexadecan-1-ol (10–25%), Geraniol (10–25%), linalool (3–5%), Nerol (3–5%), Geranyl Acetate (1–3%), Geranium oil bourbon type (1–3%), Phenethyl Acetate (1–3%), tetrahydro-4-methyl-2-(2-methylprop-1-enyl) pyran (0.1–1%), 1-(2,6,6-trimethyl-1,3-cyclohexadien-1-yl)-2-buten-1-one (<0.1%)] was chosen as it is associated with a pleasant odor in the previous literature (Ikei et al., 2015; Kroupi et al., 2016). *Mushroom* (1-Octen-3-ol; CAS number: 3391-86-4; purity: 99%, Sigma-Aldrich, United States) was chosen as it is associated with an unpleasant odor in our group’s previous study (Maharjan et al., 2019). For each of the four odors, there were three different concentrations (low, moderate, and high). Each of these concentrations was chosen after careful benchtop testing on a mixed cohort using participants from both male and female cohorts. The specific concentrations for each odor at low, moderate, and high concentrations are reported in Table 1. The flow rate was kept at 8,000 ml pm (milliliters/minute) to keep the flow rate consistent across concentrations of odors and sham stimulation. There were a total of four blocks (see Figure 1), with each block having 60-s odor delivery, 2-s class-hold time and 180-s inter-trial interval.

**TABLE 1:** Each of the odor concentrations is reported in PPM (parts per million).

Odor	Low	Moderate	High	Factor
Mushroom	11	44	175	4
Lavender	10	45	200	4.5
Jasmine	5	33	200	6
Rose	12.5	50	200	4

‘Factor’ represents the multiplication factor kept for the calculation of low, moderate, and high concentrations for each odor.

### Eye-tracker

Eye-tracker technology (Tobii Pro Glasses 2, Tobii Pro AB, Stockholm, Sweden) was used for the recording of pupillary measurements (sampling frequency, 50 Hz) that corresponds with the ANS. Before the start of the experiment, each participant’s gaze was calibrated to the glasses using a standard calibration kit provided by Tobii technologies. Once the participant’s eyes were calibrated to the device, the participant was asked to limit his or her head movement and gaze to allow for a clear and concise recording. The luminescence of the room was kept to the same level (345 *Lm*) to ensure that changes in the pupil measurements were not due to changes in light levels. During the experiments, the participants were asked to maintain their gaze on the computer throughout the whole experiment. The computer screen contained only white and black colors (black-colored text in the VAS task).

### Procedure

For this study, the male cohort completed four experimental sessions across four separate days, while the female cohort completed eight experimental sessions across 8 days, with four sessions in the follicular stage and four sessions in the luteal stage of the menstrual cycle. There was a minimum washout period of 24 h between sessions. The order of the days, and, therefore, the order of the odors and odor concentrations delivered were randomized across the participants. The experiment session started with a 5-min resting period recorded by the experimenter using a stopwatch to habituate the participant to the experiment room. After this period, each participant was fitted with ECG and eye-tracker devices. Once the devices were fitted for the participant, calibration of the participant’s gaze was performed for the eye-tracker device, which allowed the Tobii eye-tracker software to capture the participant’s gaze. This concluded the setup section of the experiment.

Each experimental session began with 4-min baseline recordings for the ECG and eye-tracker devices. The participant was seated in front of the screen at an approximately 60-cm distance, with the olfactometer breathing tube fixed just below the nostril of the participant. During the baseline recording, each participant was given a brief of the experimental procedure (Figure 1) by the experimenter, and, once the baseline time had ended, the first odor (and respective odor concentration) was delivered with an automated sequence through the olfactometer. The total experiment time for each day was approximately 24 min. There was a 4-min post-stimulation period, immediately followed by the delivery of the first odor *via* the olfactometer. After 1 min of odor delivery, this was followed by 3 min of an odor washout period. This process of odor delivery and the odor washout period was repeated four times (low, moderate, and high concentrations and one sham stimulation using distilled water). The sequence of odor delivery and inter-stimulus wash-out periods were automated using the olfactometer device. Each experimental day only contained one odor, but there were three concentrations (low, moderate, and high) of this odor per day (in addition to sham stimulation). Within the delivery of each odor (four times per session), the participants were asked to rate the odor intensity and hedonic value of the odor on separate VAS scales. These ratings were performed at roughly 25–30 and 55–60 s within the odor delivery period (1 min total). After the fourth odor washout period, the participant was instructed that a final 4-min post stimulation recording would be taken before the conclusion of the experiment. Once this post-stimulation recording was finished, each of the participants was instructed to remove the ECG and the eye-tracker device and was free to leave. On the final day of the experiment, a reimbursement voucher for the local supermarket was given.

### Data analysis

Data were averaged, extracted, and separated into the relevant experimental segments using a custom-made software in MATLAB (MathWorks, United States). Several statistical methods were used to address the research aims; all statistical analyses were completed in SPSS (Statistical Package for the Social Sciences; version 25, release 25.0.0.1, IBM Corp.). With the investigation of odor concentration effects on HRV (ECG), the pupil diameter (eye-tracker), and VAS scores, we used repeated-measures ANOVAs, observing within-subject comparisons on all three concentrations (low, moderate, and high) and sham stimulation for each odor in the inter-stimulus wash-out periods (Figure 1). Specifically for the VAS data, repeated-measure ANOVAs were conducted to observe statistical differences between the different concentrations of odors in comparison to the sham stimulation. This was performed for the intensity of the odor and the hedonic value of the odor. *Post hoc* Bonferroni tests were used to analyze pairwise comparisons from the repeated-measure ANOVA. To observe the effects of repetitive presentation of single odors (independent of odor concentration effects) on HRV and the pupil diameter, we compared the baseline period with the post-stimulation period (*see* Figure 1). Here, pairwise analysis (paired *t*-test or the Wilcoxon sign-rank test, dependent on the normality of the data) was used to analyze if there were any effects of repetitive odor stimulation on HRV and the pupil diameter. For the female cohort, these statistical methods were performed in both the follicular and luteal stages separately.

We also conducted two pilot analyses, with the data collected from this study. The first pilot analysis observed the comparison of sex and/or menstrual effects on the modulation of the ANS using olfactory stimulation. A one-way ANOVA was conducted to investigate group (male, female-follicular, female-luteal) interactions on ECG measures separately and the eye-tracker for all four odors with *post hoc* Bonferroni pairwise comparisons on any significant results. Changes between the baseline and post-stimulation periods were used for each participant. Additionally, a two-way ANOVA was used to examine the effects of ‘cohort’ (male, female-follicular, and female-luteal) and ‘concentration’ (low, moderate, and high) on HRV and pupil diameter. For each value, we used the change between sham stimulation and odor concentration for each participant. The second pilot analysis observed the interactions between the 1st washout period for each concentration of each odor and the baseline period (Figure 1) using a collated data set from both cohorts. This analysis was conducted to observe the acute effects of the first-odor presentation in comparison to the baseline in case there were any washout effects of presenting different concentrations of the same odor in the 2nd–4th presentations (Figure 1). With the randomized allocation of each participant [*n* = 21 per cohort (male, female-follicular, and female-luteal)], each presentation of odor concentration (low, moderate, high, and sham stimulation) meant that there were only five possible presentations of each odor concentration in the 1st washout period in each cohort. Therefore, we collated the data from the three cohorts to allow for *n* = 15 for this pilot observation. This was observed in both ECG domains (time and frequency).

## Results

### Visual analog scale intensity

#### Male cohort

Repeated-measures ANOVAs revealed that both the 30-s and 60-s odor intensity ratings showed significant differences between the ratings of all three concentrations of odor and sham stimulation (Supplementary Table 2, Section 1 in Appendix). The *post hoc* tests indicated significant differences between the sham stimulation and all three concentrations of each odor in the odor intensity ratings in the VAS. With regard to specific differences in odor intensity ratings of concentrations of odors, *post hoc* tests revealed significant differences across all odor intensity ratings in means of low-versus-high concentrations apart from one rating of *jasmine* at 60 s (*p*-value = 0.33). In means of odor intensity ratings of low—versus—moderate concentrations and moderate—versus—high concentrations of odors, the participants were able to distinguish *lavender* at both time points (30 s: *p*-value = 0.005; 60 s: *p*-value = 0.01) and *rose* at the 30-s time point (*p*-value = 0.024) for the former, while, in the latter, the participants were only able to distinguish in the 30-s time point for *mushroom* (*p*-value = 0.038) (Supplementary Table 2, Section 1 in Appendix).

#### Female cohort

Repeated-measures ANOVAs revealed that, in both stages of the menstrual cycle (follicular and luteal stages), for all 30s and 60-s odor ratings of intensity, there were significant differences between the ratings of all three concentrations of odor in comparison to sham stimulation (Supplementary Table 2, Section 1 in Appendix). Looking at the *post hoc* tests in all instances apart from one (female—follicular—*mushroom*—the 60-s time point: sham stimulation—versus—low concentration), women in both stages of the menstrual cycle were able to distinguish the sham stimulation from the three concentrations of the odor presentation for each odor.

In terms of detecting differences in odor intensity in separate odor concentrations of the same odor, the female participants in both stages of the menstrual cycle saw mixed results in this observation. In the follicular stage, the female cohort was able to distinguish low versus high concentrations in *mushroom* (30-s) and *jasmine* but not *lavender* and *rose.* In contrast, in the luteal stage, the female cohort could distinguish low versus high concentrations in both 30-s and 60 time points for *mushroom*, but, in only the 60-s time point for *lavender* and the 30-s time point for *jasmine* and *rose*. In the odor intensity ratings for low versus moderate concentrations, female participants in the follicular stage could distinguish *lavender* at both 30-s and 60-s time points and *rose* at the 60-s time point, while, in the luteal stage, they could distinguish *jasmine* at both 30-s and 60-s time points and *rose* at the 30-s time point. In the moderate versus high concentrations, only female participants in the luteal stage could distinguish differences in *jasmine* at the 30-s timepoint (Supplementary Table 2, Section 1 in Appendix).

### Visual analog scale hedonic value

#### Male cohort

Repeated-measures ANOVAs revealed that there were significant differences in participants’ hedonic ratings between odor concentrations and sham stimulation across all four odors, in both 30-s and 60-s time points (Supplementary Table 2, Section 1 in Appendix). According to the *post hoc* tests, in comparison to the sham stimulation, the participants were able to distinguish the *mushroom* odor as more unpleasant in the high concentrations at the 30-s time point (*p*-value = 0.046), but not in the 60-s time point. In comparison to sham stimulation, the participants rated *lavender* as significantly more pleasant in all three concentrations at both 30-s and 60-s time points. The participants also rated *jasmine* in low and high concentrations and *rose* (30-s) in moderate concentrations as more pleasant than sham stimulation. Furthermore, *post hoc* tests observing concentrations versus concentrations differences in hedonic ratings revealed that in low versus moderate comparisons, only moderate concentration of *lavender* was rated more pleasant than the low concentration at the 60-s time point. In the low versus high concentration comparisons, only *mushroom* at 30-s and 60-s time points was rated as more unpleasant in the higher concentrations. Finally, in the moderate versus high concentrations comparisons, the participants rated *mushroom* at higher concentrations as more unpleasant in both 30-s and 60-s time points (Supplementary Table 2, Section 1 in Appendix).

#### Female cohort

Repeated-measures ANOVAs (Supplementary Table 2, Section 1 in Appendix) revealed that there were significant differences between odor concentrations and sham stimulation in the luteal-menstrual stage at the 30-s time point for *jasmine* and in the follicular-menstrual stage at both time points for *lavender*. However, looking at *post hoc* tests, there were no significant differences between any of the odor concentrations and sham stimulation. None of the other comparisons of odor concentration versus sham stimulation indicated any significant differences in hedonic ratings in the female cohort at either stage of the menstrual cycle. Looking at the *post hoc* tests between hedonic ratings of different odor concentrations (low, moderate, and high), there was a significant difference in the hedonic ratings of *jasmine* in the luteal stage at the 30-s time point.

### Electrocardiogram-heart-rate variability results

#### Inter-stimulus washout period

##### Male cohort

**FIGURE 2:**
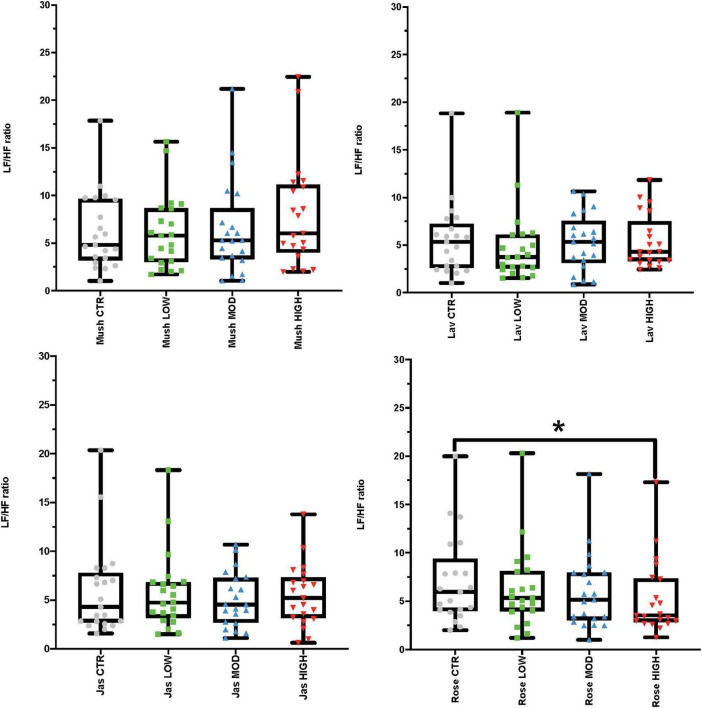
Results from the inter-stimulus wash-out periods looking at the electrocardiogram (ECG) frequency-domain analysis representing low-high frequency (LF/HF) ratio values. Each graph in the compilation represents an odor (mushroom, lavender, jasmine, and rose) and all four concentrations (sham stimulation, low, moderate-mod, and high). *p-value < 0.05 from the *post hoc* Bonferroni pairwise comparisons between sham (CTR) and high concentration.

Using repeated-measures ANOVAs, we observed the effects of each of the odor concentrations (low, moderate, and high) and sham stimulation of each odor (*mushroom, lavender, jasmine*, and *rose*) for both time and frequency domains of ECG. In the time-domain analysis, repeated-measures ANOVAs found no significant differences between RMSSD and SI scores in the comparison of each concentration of each odor against sham stimulation (Supplementary Table 3, Section 1 in Appendix). In the frequency-domain analysis, *post hoc* tests found a significant decrease in the LF/HF ratio (increase in HF in comparison to LF) after high concentration of *rose* in comparison to sham stimulation (*p*-value = 0.008, Cohen’s *D* of 0.43, indicating a small effect size) (Figure 2 and Supplementary Table 3, Section 1 in Appendix, see information (#) for this *post hoc* result in the Supplementary File, page 8). There were no other significant differences in frequency-domain measures of LF power, and HF power or LF/HF ratio measures between the sham stimulation and the three concentrations of each odor (*mushroom, lavender, jasmine, and rose*) (Supplementary Table 3, Section 1 in Appendix).

##### Female cohort

Using repeated-measures ANOVAs, we observed the effects of each of the odor concentrations (low, moderate, and high) and sham stimulation of each odor (*mushroom, lavender, jasmine*, *and rose)* for the two different domains of the ECG in both stages of the menstrual cycle. In the time-domain analysis, repeated-measures ANOVAs revealed significant differences in odor concentrations (and sham stimulation) in RMSSD scores for *jasmine* odor in the luteal stage of the menstrual cycle (*p*-value = 0.034) and SI scores for *mushroom* odor in the follicular stage of the menstrual cycle (*p*-value = 0.025) in the female participants (Supplementary Table 3, Section 1 in Appendix). However, there were no significant differences in *post hoc* tests between any of the odor concentrations and sham stimulation. In the frequency-domain analysis, repeated-measure ANOVAs revealed significant differences in odor concentrations (and sham stimulation) in LF and HF power for *rose* odor in the follicular stage of the menstrual cycle (LF power—*rose*: *p*-value = 0.043; HF power—*rose*: *p*-value = 0.022). However, *post hoc* tests indicated no significant differences between any of the *rose* odor concentrations and sham stimulation (Supplementary Table 3, Section 1 in Appendix).

#### Baseline and post-stimulation periods comparisons

##### Male cohort

**FIGURE 3:**
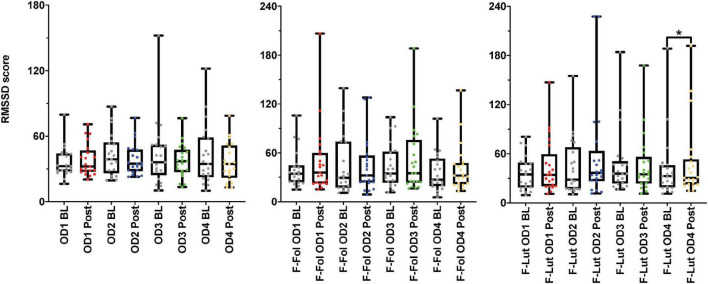
Results from the baseline (BL) and post-stimulation (Post) periods comparisons, with a focus on root mean square of successive differences between normal heartbeats (RMSSD), the electrocardiogram (ECG) time-domain parameter from both cohorts. The figure on the left represents the RMSSD results from the male cohort, while the ‘F-Fol’ represents the female cohort in the follicular-menstrual stage, while ‘F-Lut’ represents the female cohort in the luteal-menstrual stage. Each graph in the compilation represents each odor (OD1—mushroom, OD2—lavender, OD3—jasmine, OD4—rose). * – *p*-value < 0.05.

**FIGURE 4:**
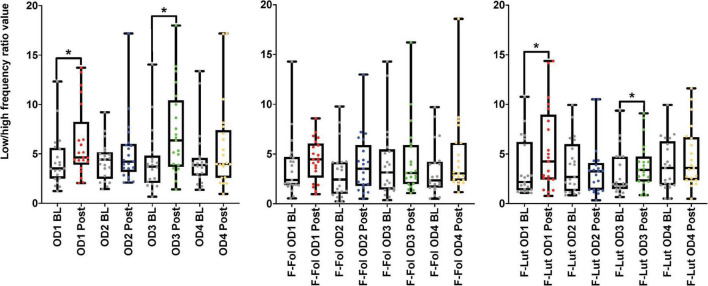
Results from the baseline (BL) and post-stimulation (Post) periods comparisons, with a focus on the low-high frequency ratio (LF/HF ratio), the electrocardiogram (ECG) frequency-domain parameter from both cohorts. The figure on the left represents the LF/HF ratio results from the male cohort, while the ‘F-Fol’ represents the female cohort in the follicular-menstrual stage, while ‘F-Lut’ represents the female cohort in the luteal-menstrual stage. Each graph in the compilation represents each odor (OD1—mushroom, OD2—lavender, OD3—jasmine, OD4—rose). * –*p*-value < 0.05.

In the observation of the baseline and post-stimulation periods comparisons, pairwise comparisons (paired *t*-tests or the Wilcoxon signed-rank test) in the ECG time domain analyses indicate no significant differences in RMSSD and SI measures in the baseline versus post-stimulation period for all four odors (*mushroom, lavender, jasmine*, *and rose*) (Figure 3 and Supplementary Table 4, Section 1 in Appendix). In the frequency-domain ECG analyses, there were significant increases in the LF/HF ratio, in the post-stimulation period in comparison to the baseline period after *mushroom* odor (*p*-value = 0.008, Cohen’s *D* of 0.58, indicating a medium effect size) and *jasmine* odor (*p*-value = 0.006, Cohen’s *D* of 0.79, indicating a medium effect size) (Figure 4 and Supplementary Table 4, Section 1 in Appendix).

##### Female cohort

Looking at baseline and post-stimulation period comparisons in the ECG time domain, only repetitive presentations of the *rose* odor were able to significantly increase RMSSD in the luteal stage of the menstrual cycle (*p*-value = 0.044, Cohen’s *D* of 0.17, indicating a small effect size) (Figure 3 and Supplementary Table 4, Section 1 in Appendix). There were no significant differences in RMSSD scores reported for the other three odors (*mushroom, lavender, or jasmine*) in the comparison of the baseline and post-stimulation periods in either stage of the menstrual cycle (Figure 3 and Supplementary Table 4, Section 1 in Appendix). There were no significant differences in all odors (*mushroom, lavender, jasmine, or rose*) for SI scores in the baseline and post-stimulation periods comparisons (Supplementary Table 4, Section 1 in Appendix). In the frequency-domain ECG analyses, there was an effect of the menstrual cycle on the effectiveness of repetitive odor stimuli to modulate the LF power and the ratio of LF and HF power (the LF/HF ratio) (Supplementary Table 4, Section 1 in Appendix). In the luteal stage of the menstrual cycle, there was a significant increase in the LF/HF ratio, with both *mushroom* (*p*-value = 0.016, Cohen’s *D* of 0.55, indicating a medium effect size) and *jasmine* (*p*-value = 0.042, Cohen’s *D* of 0.35, indicating a small effect size) odors in the baseline and post-stimulation periods comparison (Figure 4 and Supplementary Table 4, Section 1 in Appendix). These significant changes in the LF/HF ratio were not present in the follicular stage of the menstrual cycle. There were also effects of the menstrual stage on the influence of *lavender* and *rose* odors to significantly influence the LF power HRV parameter. In the follicular stage of the menstrual cycle, repetitive stimulation using the *lavender* odor causes a significant increase in LF power values (*p*-value = 0.016, Cohen’s *D* of 0.42, indicating a small effect size), whereas, in the luteal stage of the menstrual cycle, *rose* odor was able to significantly reduce the LF power (*p*-value = 0.033, Cohen’s *D* of 0.03, indicating a small effect size) in the baseline and post-stimulation period comparisons (Supplementary Table 4, Section 1 in Appendix).

### Eye-tracker-pupil diameter results

#### Inter-stimulus washout period

##### Male cohort

Repeated-measures ANOVAs looking at the inter-stimulus washout periods in pupil diameter results showed significant differences only after the *rose* odor (*p*-value = 0.04, Partial Eta squared value of 0.152, indicating a large effect size); however, after *post hoc* tests were performed, there were only significant differences between low and high concentrations (*p*-value = 0.041, Cohen’s *D*-value of 0.06, indicating a small effect size), not with sham stimulation (Table 2). No other significant differences were found between each of the odor concentrations (low, moderate or high) and sham stimulation in all four odors (*mushroom, lavender, jasmine, or rose*) (Table 2).

**TABLE 2:** Repeated-measures ANOVA results from the inter-stimulus washout period comparison for the eye-tracker (the pupil diameter) in the male and female (follicular and luteal menstrual stages) cohorts.

	Male				Female-Follicular				Female-Luteal			
	Mean	St.d.	*F*-statistic	*P*-value	Mean	St.d.	*F*-statistic	*P*-value	Mean	St.d.	*F*-statistic	*P*-value
**Mushroom**												
CTR	3.72	0.61	1.103	0.355	3.82	0.67	0.809	0.494	3.73	0.39	1.682	0.180
Low	3.74	0.62			3.82	0.64			3.76	0.39		
Mod	3.72	0.58			3.80	0.66			3.76	0.40		
High	3.74	0.61			3.83	0.63			3.74	0.38		
**Lavender**												
CTR	3.63	0.64	1.168	0.317	3.80	0.48	0.522	0.669	3.77	0.47	1.399	0.252
Low	3.66	0.60			3.79	0.48			3.75	0.44		
Mod	3.65	0.63			3.79	0.49			3.76	0.46		
High	3.64	0.65			3.80	0.46			3.76	0.46		
**Jasmine**												
CTR	3.71	0.58	0.211	0.799	3.73	0.46	1.057	0.374	3.75	0.42	0.169	0.824
Low	3.71	0.60			3.75	0.46			3.76	0.42		
Mod	3.70	0.62			3.74	0.45			3.75	0.44		
High	3.72	0.60			3.73	0.43			3.75	0.39		
**Rose**												
CTR	3.71	0.59	3.593	0.04 (0.152) *Low-high 0.041.*	3.80	0.54	0.098	0.961	3.77	0.44	2.23	0.094
Low	3.72	0.60			3.80	0.52			3.76	0.41		
Mod	3.71	0.58			3.79	0.53			3.75	0.42		
High	3.76	0.59			3.80	0.53			3.79	0.42		

All four odors (mushroom, lavender, jasmine, and rose) are reported with each concentration (sham stimulation, low, moderate-mod, and high concentrations). St.d., standard deviation. Degrees of freedom were 60. Effect sizes are presented in the ‘ANOVA’ column (partial Eta squared), and *post hoc* tests values are present in the ANOVA column in italics.

##### Female cohort

From the inter-stimulus washout periods, repeated-measures ANOVAs showed no significant differences in the pupil diameter in both stages of the menstrual cycle for all odor concentrations (low, moderate, and high concentrations) and sham stimulation for all four odors (*mushroom, lavender, jasmine, and rose*) (Table 2).

#### Baseline and post-stimulation periods comparisons

##### Male cohort

**FIGURE 5:**
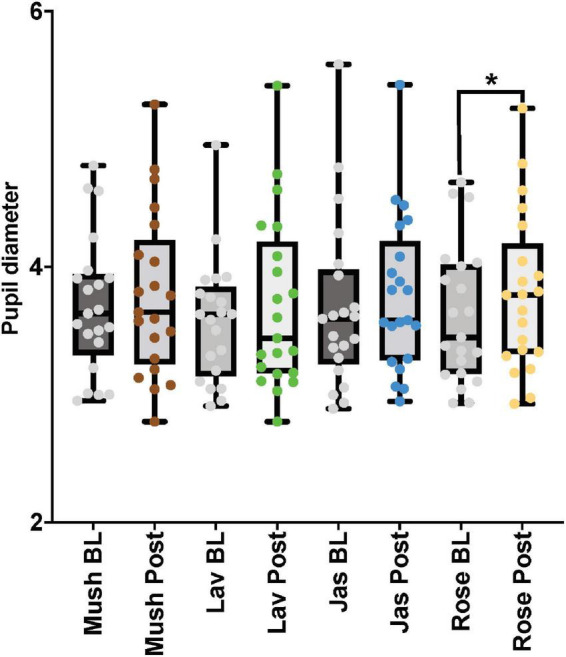
The eye-tracker (pupil diameter) results from the baseline and post-stimulation period comparisons for all four odors (Mush, mushroom; Lav, lavender; Jas, jasmine; Rose, rose) in all four concentrations (sham stimulation, low, moderate, and high) for the male cohort. *p-value < 0.05 from the paired tests comparisons.

For the paired comparisons (paired *t*-tests or Wilcoxon signed-rank tests) in the male cohort, only *rose* odor was able to increase the pupil diameter in the post-stimulation period in comparison to the baseline period (*p*-value = 0.002, Cohen’s *D*-value of 0.29, indicating a small effect size) (Figure 5 and Table 3). There were no significant differences in the paired comparison of the baseline versus post-stimulation time points for the other three odors (*lavender, jasmine, or rose*) (Figure 5 and Table 3).

**TABLE 3:** Pupil diameter results from the paired comparisons from the baseline (BL) and post-stimulation (Post) periods in the male and female (follicular and luteal menstrual stages) cohorts for all four odors (OD1— mushroom, OD2—lavender, OD3—jasmine, OD4—rose).

	Male					Female-Follicular					Female-Luteal				
	Mean	St.d.	Mean Diff.	T/Z	*P*-value	Mean	St.d.	Mean Diff.	T/Z	*P*-value	Mean	St.d.	Mean Diff.	T/Z	*P*-value
OD1 BL	3.69	0.53	−0.081	−1.31	0.202	3.72	0.71	−0.115	−2.06	0.039 (0.18)	3.66	0.41	−0.111	−2.96	0.008 (0.27)
OD1 Post	3.77	0.64				3.84	0.61				3.77	0.38			
OD2 BL	3.57	0.48	−0.119	−1.64	0.116	3.69	0.45	−0.137	−2.58	0.01 (0.30)	3.67	0.44	−0.121	−2.76	0.012 (0.25)
OD2 Post	3.69	0.67				3.83	0.47				3.79	0.46			
OD3 BL	3.67	0.66	−0.095	−1.89	0.058	3.63	0.48	−0.149	−3.00	0.003 (0.32)	3.69	0.49	−0.095	−2.05	0.053
OD3 Post	3.77	0.60				3.78	0.43				3.78	0.43			
OD4 BL	3.61	0.53	−0.169	−3.49	0.002 (0.29)	3.66	0.54	−0.186	−4.08	0.001 (0.35)	3.61	0.43	−0.192	−4.20	<0.001 (0.44)
OD4 Post	3.78	0.61				3.85	0.53				3.80	0.43			

Cohen’s *D* represented the effect size provided in brackets in the ‘*p*-value’ column. St.d., standard deviation; Mean diff., mean difference between groups; T/Z, t-statistic or Z-score. Degrees of freedom were 20.

##### Female cohort

**FIGURE 6:**
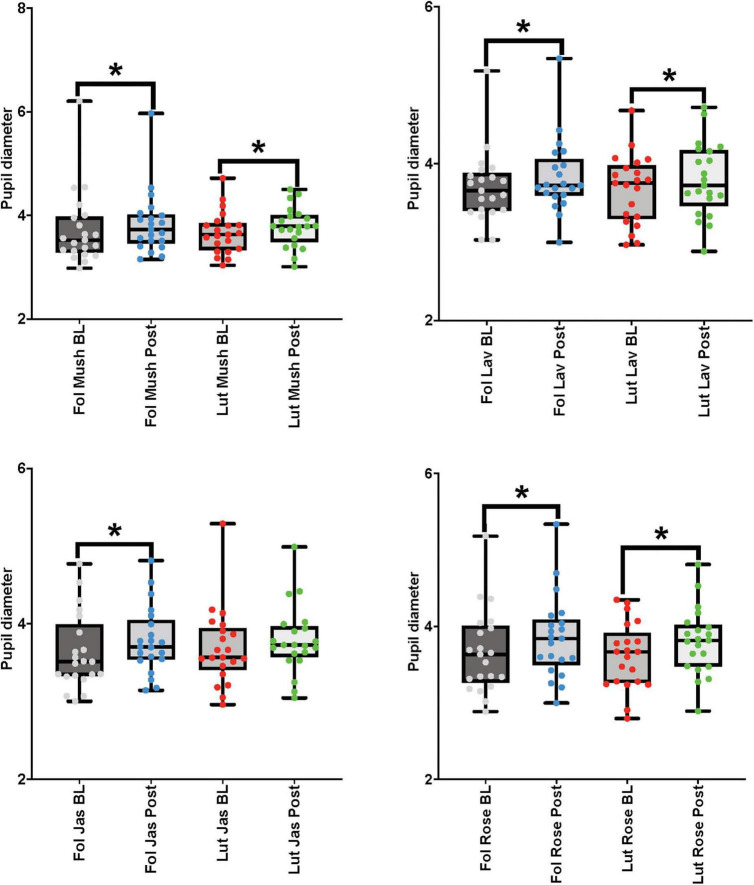
Eye-tracker (pupil diameter) results from the baseline (BL) and post-stimulation (Post) periods comparisons for all odors (Mush, mushroom; Lav, lavender; Jas, jasmine; Rose, rose) in all four concentrations (sham stimulation, low, moderate, and high concentrations) for the eye-tracker (pupil diameter) for the female cohort in both stages of the menstrual cycle (follicular-Fol and luteal-Lut). *p-value < 0.05.

For the paired comparisons (paired *t*-tests or Wilcoxon signed-rank tests) in the female cohort, after repetitive stimulation of each of the four odors, there was a significant increase in the pupil diameter in the follicular stage of the menstrual cycle in baseline and post-stimulation periods comparisons (*mushroom*: *p*-value = 0.039, Cohen’s *D*-value of 0.18, indicating a small effect size; *lavender*: *p*-value = 0.01, Cohen’s *D*-value of 0.30, indicating a small effect size; *jasmine*: *p*-value = 0.003, Cohen’s *D*-value of 0.32, indicating a small effect size; *rose*: *p*-value = 0.001, Cohen’s *D*-value of 0.35, indicating a small effect size). In comparison to the follicular stage of the menstrual cycle, in the luteal stage, repetitive stimulation of three of the four odors was able to significantly increase the pupil diameter in the baseline and post-stimulation periods comparisons (*mushroom*: *p*-value = 0.008, Cohen’s *D*-value of 0.27, indicating a small effect size; *lavender*: *p*-value = 0.012, Cohen’s *D*-value of 0.25, indicating a small effect size; *rose*: *p*-value < 0.001, Cohen’s *D*-value of 0.44, indicating a small effect size) (Figure 6 and Table 3).

#### Cohort comparisons

A one-way ANOVA revealed significant differences between the cohorts in the LF/HF ratio after *jasmine* odor in the baseline and post-stimulation period comparisons [*F* (2,60) = 4.084, *p*-value: 0.022]. *Post hoc* tests revealed that the differences lie within the male cohort and the female-follicular menstrual cohort (*p*-value = 0.026, Cohen’s *D*-value of 0.73, indicating a medium effect size), with a significantly reduced LF/HF ratio in the males in comparison to the female-follicular menstrual group (Supplementary Table A1.3, Section 2 in Appendix). Apart from this result, there were no significant differences between the cohorts in any ECG or the eye-tracker in the interaction of odors on the ANS (*see* Supplementary Tables A1.1–A1.4, Section 2 in Appendix). In addition, we observed no significant interactions between ‘cohort*concentration’ for each odor in the inter-stimulus washout period analysis (*see* Supplementary Tables A2.1–A2.6, Section 2 in Appendix).

## Discussion

In the literature, the use of both invasive (Carandina et al., 2021) and non-invasive (Li et al., 2003, 2005; Huang et al., 2005; Wu et al., 2009; Sun et al., 2013, 2015) neuromodulation techniques has shown effectiveness in the modulation of the ANS in human participants. The move to non-contact neuromodulation in comparison to these previous methods allows for easier and more natural treatment processes, and, in some cases, they have shown to be particularly effective in ANS modulation (Alaoui-Ismaili et al., 1997; Kuroda et al., 2005; Kroupi et al., 2016; Nomura et al., 2016; Tonacci et al., 2019). Although there are some explorations into the use of non-contact neuromodulation using olfactory stimulation to modulate the ANS, specific interactions of odor/s and odor concentrations and their effects are lacking in the literature. Only a couple of authors have investigated this relationship using a precise instrument of odor delivery (such as an olfactometer), with neither having investigated the role of odor concentration on the ANS (Alaoui-Ismaili et al., 1997; Nomura et al., 2016). In addition, exploration of this interaction in separate sex cohorts, which is crucial to understand the potential differences in gender effects in olfactory processing or ANS activation, is limited, with the majority of the literature exploring this relationship with a mixed cohort. In this study, we have some interesting and novel findings to highlight. (1) Non-contact neuromodulation *via* odor-olfactory stimulation could, in an acute duration study, modulate both the SNS and PNS of the ANS dependent on the sex or/and the menstrual stage (with the female cohort) of the participant. (2) There were odor presentation-specific interactions with sex and the menstrual stage on the PNS. (3) There were different ANS activation patterns measured by the ECG in comparison to the eye-tracker after the repetitive presentation of odors. (4) Certain odors can have a stimulatory effect on the SNS in the presence of a cognitive task, independent of odor concentration.

Observation of olfactory stimulation *via* both odor concentration effects and repetitive odor presentations has provided some tantalizing results on the ANS. In the male cohort, the results indicate that the use of a high concentration of *rose* odor was able to significantly increase PNS activation in comparison to sham stimulation. This was not observed when using repetitive presentation of *rose* odor in the baseline and post-stimulation periods comparisons. In contrast, the use of repetitive presentation of *rose* odor, although ineffective using a single delivery, irrespective of odor concentration, significantly increased PNS activation in the luteal-menstrual stage of the female cohort. There was also evidence to suggest that the use of repetitive *rose* odor stimulation could reduce LF power in frequency domain analyses. These results indicate that there is an odor concentration effect in the ability to stimulate the PNS network of the ANS; however, it is sex dependent. Furthermore, the modality of stimulation, acute versus repetitive, appears to also have sex- and menstrual-specific interactions on the ANS.

Results from the *jasmine* and *lavender* odors in this study *via* repetitive delivery techniques contradict some of the existing literature. Previously, some authors have concluded that the use of *lavender* and *jasmine* odors can have a sedative effect on the ANS, increasing PNS activation (Kuroda et al., 2005; Kroupi et al., 2016; Huang and Capdevila, 2017). Contrary to this, our group found that repetitive delivery of *jasmine* odor increased the LF/HF ratio toward a sympathetic dominance of the sympathovagal balance in the frequency-domain ECG. However, it is worth considering the effect of some odors, such as *jasmine*, when combined with a cognitive task. Nomura et al. (2016) found that, when *jasmine* and *lavender* odors were paired with a stressful cognitive task, it increased HR and indicated SNS dominance of the ANS. This effect also extended to just *lavender* odor alone (as a non-food odor), which is also understood to increase SNS activation when paired with a cognitive task (Tonacci et al., 2017, 2019). This is in contrast to *lavender* odor’s effects on resting-state ANS interactions (Field et al., 2005; Hongratanaworakit, 2010; Kim et al., 2013; Chen et al., 2015; Effati-Daryani et al., 2015). Therefore, this indicates that these odors could have a dichotomy of effects, depending on their presentation under resting or performing a cognitive task.

With respect to the use of non-contact, olfactory stimulation on the ANS with *mushroom* odor, this is the first time in the literature that this odor has shown SNS activation. However, it could be hypothesized that, due to the ratings (hedonic value) of the *mushroom* odor being significantly lower to that of sham stimulation, the unpleasant ratings of this odor could have resulted in the SNS response from the male participants. This is supported by existing literature that the unpleasantness of an odor is correlated with increased activity in the SNS (Tonacci et al., 2019). However, the unique result of this study extends to the modulation of SNS activity with the use of *lavender* or *jasmine* odors respective to only one stage of the female-menstrual cycle. Therefore, we build on existing literature, providing evidence that repetitive use of odors does not only interact with the state of the participant (resting or active) but with the menstrual stage in female participants. It is understood that odors can improve performances in attention and cognitive tasks through their stimulatory effects on visual tasks (Jehl et al., 1997; Larsson, 1997; Distel and Hudson, 2001; Seo et al., 2010). Therefore, it could be hypothesized that odors may increase sympathetic activity measured by the eye, and this may have resulted in an overall increase in SNS activity (independent of the menstrual stage) measured by the eye-tracker.

Although existing literature indicates that *jasmine* and *lavender* can modulate the ANS (Kuroda et al., 2005; Ikei et al., 2015; Kroupi et al., 2016; Nomura et al., 2016; Huang and Capdevila, 2017; Tonacci et al., 2019), it appears that most of the significant modulation of the ANS appears in only one domain of the ECG (the frequency domain in comparison to the time domain). It has been previously discussed that the use of ∼2–5 min of recording is optimal for frequency-domain ECG, whereas the use of >24 h is optimal for time-domain ECG analysis (Electrophysiology, 1996; Shaffer and Ginsberg, 2017). This could be the rationale for why we observed the most ANS modulation in the frequency domain, not the time domain. It has been indicated in the literature that, to correlate findings from both ECG domains, there must be 24-h whole recording for the time domain in addition to 5-min segments across the 24 h for the frequency domain (Electrophysiology, 1996). Therefore, the time allocation of the experiment could be a limiting factor in the results from the time-domain ECG in this study. Furthermore, the results from the intensity ratings in the VAS suggest that the participants were able to differentiate between the sham stimulation and each concentration of *lavender* and *jasmine* odors (low, moderate, and high). This indicates that the participants could perceive a difference in the odor presentations; however, this was not reflected in the ANS results.

It is also worth considering the method of non-contact, olfactory stimulation in this study to that of existing literature. We provided a single, acute dose of odor stimulation at different intervals or repetitive stimulation of one type of odor. There was no mixture of odors in the experimental sessions (only concentrations of the same odor). In comparison, previous literature has repeatedly used mixed odor designs. Kroupi et al. (2016) used 10 odors intermixed with 8-s odor delivery per odor. Huang and Capdevila (2017) used a mixture of citrus aurantium, linalyl acetate, linalool, and myrcene to represent *jasmine* and *lavender*, and these components were intermixed in presentation. Nomura et al. (2016) and Alaoui-Ismaili et al. (1997), although delivering odors through the olfactometer, did not segregate the presentation of odors into separate time points (or different sessions). Only one study presented odors separately; however, this study was not performed in a within-subject design (Kuroda et al., 2005). Therefore, it is not possible to confirm whether the use of a single odor led to the effects observed on the ANS in the previous literature, and, with support of the results from this study, it could be hypothesized that the mixture of the odors led to the ANS effects in the previous literature.

In the literature, there is also evidence to suggest that the subjective perception of the odor can have a significant influence on the odor’s effects on the ANS. Here, positive odors, those that are rated more pleasant, in a subjective manner, have shown the capacity to increase the activation of the PNS. In comparison, those that are rated more unpleasant, and *vice versa*, have shown the aptitude to increase the activation of the SNS (Tonacci et al., 2019). In this study, although there were significant differences between all concentrations of *lavender* (in comparison to sham) in both 30-s and 60-s ratings, there were no significant differences in the ANS responses (Supplementary File, Section 1 in Appendix). This is also true for 30-s and 60-s hedonic ratings of *jasmine* odor at low and high concentrations and 30-s *mushroom* odor at high concentrations (Supplementary File, Section 1 in Appendix). However, none of these concentrations of odors elicited a significant post-hoc response in the ANS in the inter-stimulus washout period analysis (Supplementary Table 3, Section 1 in Appendix). In contrast, *rose* odor at high concentration, which was not rated significantly different from that of sham in hedonic value (Supplementary Table 2, Section 1 in Appendix) managed to significantly increase the LF/HF ratio in post-hoc analyses, moving HRV to a parasympathetic dominance of the ANS (Supplementary Table 3, Section 1 in Appendix). There are previous reports that the use of *rose* odor is perceived as a pleasant odor and can cause feelings of comfort and relaxation in comparison to control (Igarashi et al., 2014; Tonacci et al., 2019). Although there were no differences in subjective hedonic ratings across all three concentrations of *rose* odor with the sham stimulation in luteal-menstrual period, repetitive presentation of the *rose* odor was able to increase PNS activation in the baseline versus post-stimulation periods comparison of the luteal-menstrual female cohort. The use of repetitive presentation of the *rose* odor was also able to suppress SNS activation in the baseline versus post-stimulation periods comparison in this cohort. This highlights that some odors, dependent on sex and the menstrual stage of the participant, can have an influence on the ANS irrespective of subjective or emotional influences.

With respect to the potential anatomical pathways of olfactory stimulation and its interaction with the ANS regions of the cortex, it is evident in the literature that the olfactory system has many connections with the structures of the ANS. This includes the hypothalamic, limbic, and thalamic regions, all of which contribute to the control of autonomic and behavioral arousal (Bowers, 2006). Effects of odor molecules are transmitted to the brain by olfactory sensory neurons in the nasal cavity (Bowers, 2006; Huang and Capdevila, 2017). The neuroanatomy of the olfactory system is intertwined with extensive reciprocal axonal connections with areas, such as the amygdala, hippocampus, orbito-frontal cortex, and several other regions associated with the limbic system. Orbito-frontal cortex, a hub for connections from both primary and secondary olfactory regions of the cortex (Gottfried, 2006; Patel and Pinto, 2014), is connected to the amygdala *via* GABAergic neurons, and can, therefore, cause inhibition of this structure. Activation, or disinhibition, of the central nucleus of the amygdala, which is the major efferent source of modulation of cardiovascular, autonomic, and endocrine responses, can lead to changes in HRV through several pathways. For one, activation or disinhibition of active sympathoexcitatory neurons in the rostral ventrolateral medulla *via* reduced inhibition from the caudal ventrolateral medulla and, therefore, causing an increase in SNS activation. There is also the ability to inhibit the neurons in the nucleus tractus solitarius, causing an inhibition of the nucleus ambiguus and associated dorsal motor nucleus of the vagus, which can then result in a reduction in PNS activation. There is also a potential for direct activation of sympathoexcitatory rostral ventrolateral medulla neurons that can lead to an increase in SNS activation (Saha, 2005).

In contrast to regulatory interactions of the sympathovagal activity on HRV-based ANS responses, pupillary (diameter) responses is under the control of dilator pupillae muscle (innervated *via* superior cervical sympathetic ganglion -pupillary dilation) and the sphincter pupillae muscle (innervated by the parasympathetic component of the oculomotor nerve for pupillary constriction and also inhibited by cervical sympathetic activation for early pupil dilation response) (Steinhauer and Hakerem, 1992). Both sphincter and dilator pupillae muscles control the expansion and retraction of the iris and, therefore, the diameter of the pupil *via* innervation of the cholinergic neurons of the ciliary ganglion and adrenergic neurons of the superior cervical sympathetic ganglion (Burnstock and Silito, 2000). This represents the antagonistic innervation of the two divisions of pupil responses. In combination with this, it is also known that olfactory stimulation can have a synergistic innervation to attentive and cognitive tasks, although the route of innervation from olfactory pathways to the eye is less established (Jehl et al., 1997; Larsson, 1997; Distel and Hudson, 2001; Seo et al., 2010). Understanding the innervation differences of the pupil responses in comparison to the ECG-based HRV responses helps to understand the differences in activation patterns seen after acute and repetitive delivery of *rose* odor in this study. It could be hypothesized that the odor effect, combined with the cognitive task (i.e., VAS), increased SNS activity represented in the eye; meanwhile, the vagal influence of high concentration of *rose* odor increased the parasympathetic activation in the ECG.

It is understood that there are receptors associated with gonadal hormones (such as estrogen, progesterone, and testosterone) in brain centers linked to the regulation of the ANS (Simerly et al., 1990; Liao et al., 1992; Ramirez et al., 1996). Specifically, in rodent models, Ramirez et al. (1996) found that estrogen has specific bindings sites in the ANS structures, such as the cerebellum, olfactory bulb, and the hypothalamus. It is evident that there is an influence of menstrual (and sex) hormones, such as estrogen, progesterone, and testosterone in brain regions associated with the control of the ANS. Their role in these structures (such as the hypothalamus, nucleus tractus solitarius, amygdala, hippocampus, cerebellum, and limbic structures) is often to modulate each other’s role, which, in some cases, are antagonistic (Gruber et al., 2002; Brinton et al., 2008; Jacobs and D’Esposito, 2011). Unlike research into estrogen, progesterone has only been recently received attention to its role in the ANS of the brain. The role of progesterone in the ANS is highlighted by its appearance in key structures that mediate the ANS, such as the amygdala, nucleus tractus solitarius, rostral ventrolateral medulla, hypothalamus, cerebellum, and hippocampus (Auger and De Vries, 2002; Saha, 2005; Brinton et al., 2008). There are already highlighted differences found between estrogen and progesterone in rodent studies, where it appears that the developmental age causes a different pattern of appearance on these hormones. Although it has been previously perceived that the role of progesterone was to mediate antagonistic actions toward estrogen, more recent research has suggested that progesterone could also have antiapoptotic, neuroregenerative, and neuroprotective effects in the brain (Brinton et al., 2008).

It has previously been established in animal models that the use of intravenous administration of estrogen can increase vagal tone and suppress SNS activity (Saleh and Connell, 1999; Saleh et al., 2000). This effect could have masked a potential increase of the folicular menstrual-stage LF/HF ratio that is found in the luteal-menstrual stage *via mushroom* and *jasmine* odors. As we still found increased LF power with *lavender* in the follicular-menstrual stage, in addition to an increased SNS activation in the pupil-ANS data, this suggests that some odors could have interactions with specific menstrual hormones such as estrogen. It is also unclear whether the interaction of odors on the ANS could be mediated *via* progesterone in the luteal-menstrual stage. Such conclusions require future research on the interactions between odor stimulation and menstrual hormones. Reduced PNS activation has also been associated with the luteal stage of the menstrual cycle (Matsumoto et al., 2006, 2007, 2013a; Baker et al., 2008). To remedy this, some authors have used *lavender* odor to increase PNS activity in this stage of the menstrual cycle (Matsumoto et al., 2013b). Although we did not observe any significant effects of *lavender* odor (after acute—single concentration or repetitive presentations) in modulating PNS activity, we did, however, find an increase in PNS activation (RMSSD) after the repetitive presentation of *rose* odor in the luteal-menstrual stage. What is fascinating about this result is that, in comparison to the result from Matsumoto et al. (2013b), the average baseline score of RMSSD in the luteal-menstrual stage was at the same level as that of post-stimulation levels in the follicular stage. Despite this, the use of repetitive delivery of *rose* odor was still able to significantly increase RMSSD scores in the post-stimulation period in comparison to the baseline period. Therefore, this result indicates that repetitive delivery of *rose* odor could be more powerful than that of *lavender* odor to increase PNS activation in the luteal-menstrual stage.

The clinical applications of HRV measures are closely tied to several health conditions, such as chronic inflammatory disorders and work-related stress. The overarching issue is the imbalance of sympathovagal innervation toward sympathetic dominance (reduced vagal activation) (Friedman et al., 1993; Electrophysiology, 1996; Friedman and Thayer, 1998a,b; Srinivasan et al., 2002; Limm et al., 2010; Shin and Liberzon, 2010; Taelman et al., 2011; Shaffer and Ginsberg, 2017). Several chronic inflammatory disorders, such as rheumatoid arthritis, and inflammatory bowel diseases, present a reduced vagal-parasympathetic activation of the sympathovagal balance, and, due to chronic inflammation (persistent and unregulated TNF-α levels), there is a significant amount of chronic tissue damage, which is the driver for the pathogenesis of these conditions (Bonaz et al., 2016; Kalliolias and Ivashkiv, 2016; Koopman et al., 2016; Tsaava et al., 2020). Workplace-related stress, which accounts for ∼40–50% of all work-related absences, costs businesses billions of dollars year (Limm et al., 2010; Taelman et al., 2011). This form of stress is interlinked with increase in population prevalence of chronic inflammatory disorders (Limm et al., 2010; Taelman et al., 2011). There is evidence in the literature that suggests that the use of certain odors can help reduce work-related stress (Chang and Shen, 2011; Souto-Maior et al., 2011). These odors help improve performances in computerized tasks and help to reduce sympathetic activation in combination with increasing parasympathetic-vagal activity (Huang and Capdevila, 2017). From the results of this article, two different rose odor delivery methods are proposed to improve vagal-parasympathetic tone in the aforementioned health conditions, dependent on sex of the patient. For male patients, the use of a short duration of high-concentration *rose* odor, while, for female patients, with respect to the luteal-menstrual stage, the use of long-term *rose* odor presentation.

With the prevalence of COVID-19 throughout the last few years, there is a growing need to focus on the sympathovagal balance of the ANS. The consequence of getting a coronavirus disease-2019 (COVID-19) infection for sympathovagal balance, irrespective of age, is a cause for concern (Barber, 2020). Upregulation of circulating cytokines is particularly important to strengthen the immune system during viral infection (Tsaava et al., 2020). It is well understood that stimulation of the vagal tone provided through vagus nerve stimulation can assist in cytokine regulation in chronic inflammatory disorders (Bonaz et al., 2016; Kalliolias and Ivashkiv, 2016; Koopman et al., 2016; Tsaava et al., 2020). During an immune response, vagus nerve stimulation can help suppress cytokine release in the context of chronic inflammation (Tsaava et al., 2020). Of particular importance is the research on severe acute respiratory syndrome coronavirus-2 (SARS-CoV-2), where this viral infection triggers an excessive immune response known as a ‘cytokine storm’ (Hu et al., 2021). The cytokine storm has the potential to become a fatal immune disease that is characterized by a high level of immune cell activation and extremely elevated production of inflammatory cytokines and chemical mediators (Teijaro et al., 2014). This effect has been recently attributed to the main cause of disease severity and death in patients with COVID-19 (Mehta et al., 2020). Regulation of cytokine response *via* the immune system is integral to combating this viral influence. There is potential for the regulation of cytokine response to be mediated through the use of peripheral stimulation techniques that can activate the vagus network (Bonaz et al., 2016; Kalliolias and Ivashkiv, 2016; Koopman et al., 2016; Genovese et al., 2020; Marsal et al., 2021). Therefore, there is a potential for stimulation of the vagal tone *via* specific odors, such as *rose*, to work as a neuroprotective treatment before or during viral infections, such as COVID-19.

Despite novel findings in this study, it is not without some limitations. One is the recording duration of the session, i.e., post-odor delivery effects. Our study used 1-min odor delivery, which was within the range (from 8 s to 30 min) of odor delivery in the existing literature (Alaoui-Ismaili et al., 1997; Kuroda et al., 2005; Ikei et al., 2015; Kroupi et al., 2016; Nomura et al., 2016; Huang and Capdevila, 2017; Tonacci et al., 2019). The use of 1-min odor delivery also reduced odor habituation, and, in fact, there is no established standard to the amount of time to present an odor to influence the ANS in the literature. This also means that the effects found with ANS modulation *via* odor/s or specific concentrations of odor/s are only of an acute nature, and a chronic study design over a longer duration would be required to understand the long-term effects of odors and specific odor concentrations on the ANS. A second limitation pertains to the menstrual stages observed in the female cohort. We did so following previous protocols used to classify the menstrual cycle into the two main menstrual stages (follicular and luteal) (Derntl et al., 2013). Future research could include menstrual periods, such as menses and days of/around ovulation. The menstrual period of ovulation (Le Magnen, 1952; Vierling and Rock, 1967; Mair et al., 1978; Doty et al., 1981) and menses (Le Magnen, 1952; Schneider and Wolf, 1955; Good et al., 1976; Mair et al., 1978; Moriyama and Kurahashi, 2000) are indicated to cause significant differences in olfactory processing and ANS activation in comparison to the follicular and luteal stages. Finally, the current study used a small sample size of *n* = 21 per cohort. The sample size was based on the results from our previous study (Maharjan et al., 2021), and, due to the study being of within design, the *n* was sufficient to obtain an appropriate effect size for our results. However, future replications should increase the *n* to get a stronger power and effect size in the results.

We also would like to acknowledge that the significant post-hoc result of the Male cohort for high concentration versus sham control of Rose odour on LF/HF ratio has been obtained when ANOVA result were not significant for Control-Low-Moderate-High analyses for LF/HF ratio of Rose odour in Male cohort (Supplementary Table 4, Section 1 in Appendix). While the common practice is not to proceed with post-hoc tests when ANOVA results is not significant, there are also statisticians that considers that it’s unfortunate to run multiple comparisons when only the null hypothesis of homogeneity is rejected (https://www.graphpad.com/support/faqid/1081/). Since the post-hoc tests are focused, it is postulated that they have the power to find differences between groups even the overall ANOVA is not significant and such post-hoc results are considered as valid (https://www.graphpad.com/support/faqid/1081/). In the context of above mentioned aspects and considering the investigatory scope of the present study, we included the significant post-hoc results for High-concentration rose on LF/HF ratio in Male Cohort by acknowledgment of the practice.

Finally, it is worth briefly discussing the two pilot analyses performed in this study. The first was to observe cohort comparisons for both results from the inter-stimulus washout period and baseline versus post-stimulation period comparisons. The second was the observation of acute (∼8 min) effects of each odor concentration in comparison to the baseline period (*see* Supplementary Appendix, Section 3). With the cohort comparison, there were only significant differences in SNS dominance of the LF/HF ratio between the male and female follicular menstrual cohorts (*see* Supplementary Appendix, Section 2). There were no significant differences in the menstrual stage comparisons. These results indicate that, although SNS dominance (increased LF/HF ratio) was significantly increased in the luteal-menstrual cohort after the repetitive presentation of *jasmine* odor when observing the cohorts separately (Supplementary Table 4, Section 1 in Appendix), in the comparison of the cohorts, significant LF/HF ratio differences between sex only occurred in the male versus the female follicular menstrual group (*see* Supplementary Appendix, Section 2). This would suggest that, although there was increased SNS activation (over PNS) in the female-luteal cohort after repetitive *jasmine* odor presentations, comparison of cohorts indicates that significant differences were only present between the female follicular and male cohorts (particularly because of the profound and significant effect of *jasmine* on LF/HF ratio in male cohort and minimal effect of *jasmine* on LF/HF ratio in the follicular stage). There were no other significant differences in the cohort comparisons. It is apparent that, with the change of analysis from a within-subject to a between-subject design, the current sample size is not sufficient (A sample size of ∼66 participants per cohort would be sufficient for between-subject design). Using a mixed-model ANOVA, where fixed variables included odors (mushroom, lavender, jasmine, and rose), cohort (male, female-follicular, and female-luteal), and odor concentration (delta of low, moderate, and high in comparison to sham), across all ECG and eye-tracker variables, we found no significant differences in RMSSD (*F*-statistic: 0.492, *p*-value = 0.92), SI (*F*-statistic: 0.182, *p*-value = 0.999), LF-power (*F*-statistic: 0.294, *p*-value = 0.99), HF-power (*F*-statistic: 0.64, *p*-value = 0.808), the LF/HF ratio (*F*-statistic: 0.274, *p*-value = 0.993), or pupil dilation (*F*-statistic: 0.506, *p*-value = 0.911). The primary aim of this study was to observe the acute effects of odor concentration on the ANS. Additional observations using between-subject analysis change the study design, and, therefore, we require a large sample size to fulfill these investigations. With the second pilot analysis observing the effects of acute, single odor (1st odour presentation with respective concentration) effects in comparison to the baseline period in the collated data set of all cohorts (Supplementary Appendix, Section 3), a high concentration of *rose* odor was able to increase SNS activation in the time-domain ECG. This was in contrast to the high concentration of *rose* odor, increasing PNS activation in the frequency-domain ECG in the male cohort (Supplementary Table 3, Section 1 in Appendix). It appears that combining the cohorts could show very contrasting effects on the ANS. This highlights the importance of observing cohorts (sex and the menstrual stage) separately. However, results from both the cohort comparison and the acute pilot observation of one odor effect should be interpreted with certain limits. The main focus of this study was on the investigation of olfactory stimulation effects on the ANS, controlling for the sex and the menstrual stage of the participants. Our goal was to avoid the use of a mixed-sex (and menstrual stages) cohort as results could potentially be blind to significant changes apparent in only one cohort. In particular, there is insufficient *n* for these pilot analyses, and future exploration of these pilot analyses should be performed with a larger population group that facilitates a between-subject design and a simplified experiment with just one stimulation per session.

In conclusion, for the first time in olfactory stimulation research on the ANS, we found acute odor-specific, concentration-specific, and odor delivery-specific effects of odors on the PNS activation respective to sex and the menstrual stage *via* non-contact neuromodulation. While a high concentration of *rose* odor was able to increase the shift of ANS activation toward a parasympathetic dominance in the males (Supplementary Table 3, Section 1 in Appendix), repetitive presentation of the same odor improved PNS activation in the females in only the luteal stage of the menstrual cycle (Figure 3 and Supplementary Table 4, Section 1 in Appendix). Furthermore, the effects of specific odors, such as *mushroom*, *lavender*, and jasmine, on the sympathovagal activity based ANS activity (HRV), given in a repetitive manner, were menstrual stage specific in the female participants (Supplementary Table 4, Section 1 in Appendix). This was in contrast to the effects of these odors on the oculomotor/cervical sympathetic nerves-dependent ANS activity (pupil diameter), which occurred in both menstrual stages (except Jasmine for Folicular stage only, Table 3). We also found that *mushroom* and *jasmine* odors, irrespective of sex but respective to the menstrual stage in the female participants, when paired with a cognitive task and given in a repetitive manner, will increase the sympathetic modulation of the ANS (Figure 4 and Supplementary Table 4, Section 1 in Appendix). Modulation of the sympathovagal balance *via* vagal-parasympathetic modulation is crucial in the field of chronic inflammatory disorders. The research in the current study highlighted the importance of stimulation parameters of olfactory stimulation, such as duration, frequency, and strength. These measures should be of particular focus when observing the interactions of olfactory stimulation on the ANS in combination with sex- and menstrual stage-specific research using separate cohorts to account for sex.

## Data availability statement

The raw data supporting the conclusion of this article will be made available by the authors, without undue reservation (conditional to ethical approval restrictions).

## Ethics statement

The studies involving human participants were reviewed and approved by the Otago Human Participants Ethics Committee (reference: H20/123) and the Australian New Zealand Clinical Trials Registry (ANZCTR number: 12622000415707). The patients/participants provided their written informed consent to participate in this study.

## Author contributions

YC, MP, and AM contributed to project design, statistical analysis, and interpretation of the results. AM contributed to performing experiments. AM and PK contributed to data collection. YC, MP, AM, and PK contributed to finalizing the manuscript. All authors contributed to the article and approved the submitted version.
