# Supplementary material for: Non-contact neuromodulation of the human autonomic nervous system function via different odors: Sex, menstrual cycle, and odor dose- and duration-specific effects
Source: Front Neurosci. 2022 Oct 12;16:950282. doi: 10.3389/fnins.2022.950282 (PMC9596915; doi:10.3389/fnins.2022.950282)
Supplement: Supplementary file 1 [file Data_Sheet_1.pdf]

Supplementary file - Article: Non-contact neuromodulation of the human autonomic nervous system function via different odors: Sex, menstrual cycle, and odor dose- andduration-specific effects

Appendix

Section 1

Table 2. Results from the repeated-measures ANOVAs looking at the visual analogue scale (VAS) scores for ‘Intensity’ and ‘Hedonic value’ ratings in all four concentrations (low, moderate-mod and high, sham stimulation-CTR) of each odour of both male and female (follicular and luteal menstrual stages) cohorts. Posthoc Bonferroni pairwise comparisons were performed for each repeated-measures ANOVAs to observe differences across odour of different concentrations (and sham). Posthoc comparisons are coded ‘i’ - CTR-low, ‘ii’ - CTR-mod, ‘iii’ - CTR-high, ‘iv’ - low-mod, ‘v’ - low-high, ‘vi’ - mod-high. The ratings for 30 s and 60 s were also divided into two sections. Partial Eta squared represented effect size, which is provided in the ANOVA columns inside brackets. St.d. = standard deviation, Bonf. = Bonferroni. Degrees of freedom - 3, 60.

Male										
	Intensity 30s					Intensity 60s				
	**Mean**	**St.d.**	**F-statistic**	**p-value**	**Posthoc**	**Mean**	**St.d.**	**F-statistic**	**p-value**	**Posthoc**
**Mushroom**										
CTR	9.42	10.39	38.588	0.000 (0.659)	i- 0.02, ii & iii- <0.001, iv-0.113, v-0.003, vi-0.038.	9.52	12.19	26.591	0.000 (0.571)	i- 0.006, ii & iii- <0.001, iv-0.245, v-0.002, vi-0.157.
Low	35.90	24.47				29.52	20.37			
Mod	46.57	19.62				38.42	18.54			
High	60.19	17.92				50.09	24			
**Lavender**										
CTR	6.66	7.52	53.235	0.000 (0.727)	i, ii & iii- <0.001, iv-0.005, v-0.002, vi-1.	7.14	8.29	39.743	0.000 (0.665)	i, ii & iii- <0.001, iv-0.01, v-0.004, vi-1.
Low	34.09	19.28				27.42	16.83			
Mod	53.28	18.52				45.61	17.54			
High	53.28	18.13				46.28	15.88			
**Jasmine**										
CTR	11.42	21.34	20.707	0.000 (0.509)	i & iii- <0.001, ii-0.003, iv-1, v-0.024, vi-0.273.	10.57	15.42	15.222	0.000 (0.432)	i- 0.001, ii-0.003, iii- <0.001, iv-1, v-0.33, vi-0.439.
Low	38.42	16.53				34.57	20.37			
Mod	43.14	20.84				35.85	21.07			

High	54.19	18.90				45.90	19.41			
**Rose**										
CTR	8.80	10.24	53.195	0.000 (0.727)	i, ii & iii- <0.001, iv-0.024, v-<0.001, vi-0.203.	4.85	5.55	48.761	0.000 (0.709)	i, ii & iii- <0.001, iv-0.066, v-0.002, vi-1.
Low	37.09	16.87				32.57	18.87			
Mod	51.52	18.04				46	18.75			
High	61.14	22.40				50.52	16.77			
	**Hedonic value 30s**					**Hedonic value 60s**				
	**Mean**	**St.d.**	**F-statistic**	**p-value**	**Posthoc**	**Mean**	**St.d.**	**F-statistic**	**p-value**	**Posthoc**
**Mushroom**										
CTR	6.04	19.08	6.86	0.000 (0.255)	iii-0.046, v-<0.001, vi-0.003.	1.19	30.30	4.239	0.024 (0.175)	v-0.002, vi-0.014.
Low	8.95	36.05				5.76	33.19			
Mod	1.76	34.96				-1.66	28.43			
High	-17.71	34.63				-16.71	34.01			
**Lavender**										
CTR	3.04	7.87	11.493	0.000 (0.365)	i & ii-<0.001, iii-0.003	-0.429	13.27	13.97	0.000 (0.411)	i-0.004, ii-<0.001, iii-0.001, iv-0.039.
Low	31.09	23.06				20.857	18.15			
Mod	34.09	26.04				33.19	23.81			
High	27.95	25.05				24.95	20.81			
**Jasmine**										
CTR	6.95	18.39	5.739	0.002 (0.223)	i-0.006, iii-0.014.	7.19	18.10	5.893	0.001 (0.228)	i-0.002, iii-0.024.
Low	25.61	22.94				26.95	22.27			
Mod	24.09	16.51				22.28	18.43			
High	28.14	31.68				25.90	30.12			
**Rose**										
CTR	2.71	16.35	5.14	0.003 (0.204)	ii-0.028.	3.42	19.10	4.03	0.011 (0.168)	
Low	24.42	33.79				22.42	35.85			
Mod	27.81	36.50				24.57	34.72			
High	26.95	40.58				26.38	41.24			
**Female-Follicular**										
	**Intensity 30s**					**Intensity 60s**				
	**Mean**	**St.d.**	**F-statistic**	**p-value**	**Posthoc**	**Mean**	**St.d.**	**F-statistic**	**p-value**	**Posthoc**
**Mushroom**										
CTR	25.04	28.39	17.213	0.000 (0.463)	i- 0.041, ii & iii- <0.001, iv-0.123, v-0.011, vi-1.	19.66	24.50	12.551	0.000 (0.386)	i- 0.063; ii & iii- <0.001, iv-0.265, v-0.079, vi-1.
Low	42.28	24.48				35.57	25.90			
Mod	55.42	24.48				47.14	27.87			
High	61.95	28.82				53.04	27.74			
**Lavender**										

CTR	11.95	15.84	29.609	0.000	i, ii & iii- <0.001,	10.04	15.34	19.443	0.000	i-0.001, ii & iii- <0.001,
Low	37.80	17.81		(0.597)	iv-0.002, v-0.146,	27.19	20.37		(0.493)	iv-0.012, v-0.068,
Mod	52.61	19.12			vi-1.	41.71	22.62			vi-1.
High	53	28.05				43.71	26.64			
**Jasmine**										
CTR	18.09	21.70	36.896	0.000	i, ii & iii- <0.001,	15.85	19.61	38.085	0.000	i, ii & iii-<0.001, iv-1,
Low	49.23	32.81		(0.648)	iv-0.232, v-0.026,	46.33	27.65		(0.656)	v-0.034, vi-1.
Mod	59.28	28.49			vi-1.	50.90	28.83			
High	62.80	29.24				55.57	25.68			
**Rose**										
CTR	15.95	17.91	37.519	0.000	i, ii & iii- <0.001,	14.23	18.77	30.424	0.000	i-0.001, ii & iii- <0.001,
Low	52.14	29.42		(0.652)	iv-0.449, v-0.113,	42.52	29.52		(0.603)	iv-0.03, v-0.082,
Mod	61.38	24.19			vi-1.	54.95	26.36			vi-1.
High	65.61	28.73				56.04	27.46			
	**Hedonic value 30s**					**Hedonic value 60s**				
	**Mean**	**St.d.**	**F-statistic**	**p-value**	**Posthoc**	**Mean**	**St.d.**	**F-statistic**	**p-value**	**Posthoc**
**Mushroom**										
CTR	19.80	25.02	0.903	0.445		15.04	23.14	1.148	0.337	
Low	15.71	31.77				9.09	26.78			
Mod	10.57	45				15.85	34.73			
High	6.71	38.54				4.80	32.05			
**Lavender**										
CTR	11.90	27.59	4.010	0.027		9.90	24.06	4.297	0.018	
Low	26.19	27.19		(0.167)		19.95	21.87		(0.177)	
Mod	32.76	35.07				28.90	31.45			
High	29.23	34.48				32.76	32.76			
**Jasmine**										
CTR	17.47	30.77	1.143	0.323		14.09	29.92	0.743	0.477	
Low	25.09	37.52				23.47	31.33			
Mod	25.47	48.61				22.80	46.6			
High	12.57	51.22				14.19	50			
**Rose**										
CTR	15.23	24.47	2.559	0.086		12.52	21.43	1.780	0.161	
Low	37.76	45.01				31.47	41.49			
Mod	27.33	40.44				21.28	41.47			
High	24.47	46.35				20.42	40.89			
**Female-Luteal**										

	Intensity 30s					Intensity 60s				
	**Mean**	**St.d.**	**F-statistic**	**p-value**	**Posthoc**	**Mean**	**St.d.**	**F-statistic**	**p-value**	**Posthoc**
**Mushroom**										
CTR	19.38	20.94	26.160	0.000 (0.567)	i- 0.002; ii & iii- <0.001; iv-0.691, v-0.005, vi-0.109.	15.09	18.43	20.275	0.000 (0.503)	i- 0.001; ii & iii- <0.001; iv-1, v-0.019, vi-1.
Low	45	25.91				41.09	26.94			
Mod	53.66	23.60				45.80	26.71			
High	68.14	29.50				58.85	25.68			
**Lavender**										
CTR	18.90	23.59	25.104	0.000 (0.557)	i, ii & iii- <0.001; iv-1, v-0.207, vi-1.	17.10	23.33	19.024	0.000 (0.500)	i-0.004, ii-0.001, iii-<0.001, iv-1, v-0.008, vi-0.460.
Low	48.66	29.14				39.65	32.02			
Mod	51.61	23.28				44.7	26.35			
High	57.09	26.30				54.05	29.99			
**Jasmine**										
CTR	10.71	17.81	57.092	0.000 (0.741)	i, ii & iii- <0.001, iv-0.025, v-0.002, vi-0.039.	12.42	21.30	28.785	0.000 (0.590)	i, ii-& iii-<0.001; iv-0.042, v-0.339, vi-1.
Low	43.57	24.12				40.23	21.11			
Mod	57.09	22.64				52.42	24.90			
High	65.71	21.37				51.19	22.72			
**Rose**										
CTR	14.14	15.43	51.764	0.000 (0.721)	i, ii & iii- <0.001; iv-0.037, v-0.045, vi-1.	11.52	16.42	41.110	0.000 (0.673)	i, ii & iii- <0.001; iv-1, v-0.56, vi-1.
Low	52.85	22.09				50.47	23.39			
Mod	64.09	23.60				54.80	21.44			
High	69.23	25.85				59.09	22.48			
	**Hedonic value 30s**					**Hedonic value 60s**				
	**Mean**	**St.d.**	**F-statistic**	**p-value**	**Posthoc**	**Mean**	**St.d.**	**F-statistic**	**p-value**	**Posthoc**
**Mushroom**										
CTR	21.66	29.12	1.682	0.201		14.52	29.55	1.300	0.284	
Low	16.23	37.2				13.33	36.26			
Mod	23.80	34.12				18.71	33.81			
High	6.57	49.39				5.33	41.87			
**Lavender**										
CTR	15.80	33.48	1.234	0.305		13.23	37.60	0.605	0.615	
Low	30	44.35				17	43.13			
Mod	28.61	36.28				20.80	36.12			
High	29.04	42.29				21.95	41.36			
**Jasmine**										
CTR	11.09	22.69	3.637	0.038 (0.154)	vi-0.021.	12.47	24.81	3.149	0.052	
Low	27.85	40.86				23.71	40.59			

Mod	32.33	46.81				28.47	46			
High	13.76	46.25				7.66	37.15			
**Rose**										
CTR	10.23	31.60	2.433	0.109		8.95	27.87	0.962	0.396	
Low	31.57	38.87				18.09	36.35			
Mod	26.38	47.08				22.09	47.29			
High	11.14	45.71				9.28	40.92			

Table 3. Results from the repeated-measures ANOVAs from the inter-stimulus wash-out periods looking at the electrocardiogram (ECG) time and frequency domains. For time-domain, results from root mean square of successive differences between normal heartbeats (RMSSD) and stress index (SI) are presented. For frequency-domain, results from low-frequency (LF) power, high-frequency (HF) power and low-high frequency ratio (LF/HF ratio). The values of all four concentrations (low, moderate-mod and high concentrations, sham stimulation - CTR) of each odour are presented. Posthoc Bonferroni pairwise comparisons were performed for each repeated-measures ANOVAs to observe differences between each concentration of odour (and sham). Posthoc-iii = Sham stimulation versus high concentration. Effect sizes are reported in brackets on significant p-values and represents partial Eta squared unless stated otherwise. St.d. = standard deviation. Degrees of freedom was 3, 60.

	**Male**				**Female-Follicular**				**Female-Luteal**			
	**Mean**	**St.d.**	**F-statistic**	**p-value**	**Mean**	**St.d.**	**F-statistic**	**p-value**	**Mean**	**St.d.**	**F-statistic**	**p-value**
**RMSSD**												
**Mushroom**												
CTR	33.07	10.90	1.278	0.290	35.27	24.61	2.298	0.147	36.95	19.50	0.798	0.458
Low	35.89	13.68			34.50	22.32			36.38	20.31		
Mod	35.96	14.96			36.43	19.21			34.81	17.18		
High	35.64	12.24			39.36	23.27			37.09	21.01		
**Lavender**												
CTR	35.74	15.89	0.092	0.964	42.63	34.76	0.543	0.633	37.05	26.54	2.495	0.077
Low	36.29	13.09			40.68	28.56			35.22	17.59		
Mod	36.68	13.31			41.64	34.71			32.32	19.04		
High	36.44	11.81			43.25	31.50			40.88	26.37		
**Jasmine**												

CTR	35.17	13.39	1.062	0.372	40.80	27.82	0.172	0.840	35.29	20.92	3.850	0.034 (0.161)
Low	35.68	15.43			39.44	21.99			33.66	21.16		
Mod	35.98	13.81			39.57	21.82			36.18	21.42		
High	38.79	22.86			40.14	25.30			38.61	23.54		
**Rose**												
CTR	33.20	13.31	1.814	0.154	36.74	25.62	0.925	0.434	39.01	29.12	1.160	0.307
Low	32.98	14.26			35.70	19.93			46.44	54.82		
Mod	35.71	16.36			34.76	22.47			43.23	41.15		
High	35.20	15.45			33.90	19.27			43.90	44.47		
**Stress index**												
**Mushroom**												
CTR	9.77	2.18	0.829	0.483	11.70	4.27	3.329	0.025 (0.143)	11.10	4.39	0.146	0.830
Low	9.23	2.64			11.64	4.10			11.26	4.95		
Mod	9.53	2.70			10.34	2.68			11.32	4.80		
High	9.33	2.33			10.83	3.66			10.94	4.55		
**Lavender**												
CTR	9.86	3.24	0.377	0.770	11.47	5.08	0.665	0.532	11.91	4.09	2.738	0.051
Low	9.39	2.74			11.77	5.90			10.84	4.02		
Mod	9.63	3.02			11.24	4.63			12.15	4.18		
High	9.57	3.06			10.85	4.75			10.40	3.81		
**Jasmine**												
CTR	10.12	3.77	0.537	0.659	10.28	3.71	0.746	0.489	11.63	4.05	1.523	0.218
Low	10.42	4.01			10.87	3.91			11.54	3.37		
Mod	9.98	3.69			10.44	3.61			11.26	3.97		
High	9.95	4.05			10.91	5.16			10.75	4.46		
**Rose**												
CTR	10.32	3.37	0.333	0.802	11.67	4.31	0.938	0.645	11.08	3.65	0.067	0.977
Low	10.59	3.85			11.31	4.20			10.93	3.96		
Mod	10.15	3.74			12.05	5.41			11.10	4.70		
High	10.56	4.20			11.94	4.29			10.91	3.97		
**Low-frequency power**												

**Mushroom**												
CTR	2051.8	1251.2	1.109	0.353	1843.5	1242.3	0.508	0.678	2041.7	1783.3	0.517	0.672
Low	2347.1	1486.4			1615.5	1376.7			1708.5	1216.6		
Mod	2156.2	1044.2			1613.8	1000.2			1992.8	1577.9		
High	2512.9	1658.7			1593.7	1433.6			1998.3	1468.9		
**Lavender**												
CTR	2010.7	1122.8	1.487	0.227	2143.8	1888.1	0.275	0.843	1385.6	1093.8	3.091	0.051
Low	1894	1193			2079.2	2060.0			1671.8	1163.5		
Mod	2352.7	1495.6			2381.9	2227.4			1480.3	962.8		
High	2417.1	1426.6			2256.5	1731.3			1916.7	1236.0		
**Jasmine**												
CTR	2191.1	1399.2	0.371	0.774	2142.0	2534.1	1.335	0.270	1780	1447.0	0.590	0.565
Low	2084.4	1197.8			1472.0	905.6			1668.1	1390.0		
Mod	2242.5	1531.7			1487.5	694.4			1812.1	1365.0		
High	2376.6	2420.9			1642.6	1424.3			2035.1	1955.4		
**Rose**												
CTR	2220.5	1492.4	0.34	0.796	1571.0	1459.9	2.875	0.043 (0.126)	1704.9	1493.1	0.223	0.789
Low	2030.7	1438.6			1329.4	1008.3			1886.0	1985.2		
Mod	2177.7	1313.1			1889.7	1465.5			1791.3	1417.3		
High	2370.9	2086.9			1221.9	951.2			1995.0	2037.6		
**High-frequency power**												
**Mushroom**												
CTR	434.52	361.3	0.655	0.583	622.42	905.88	0.416	0.621	647.52	576.19	0.138	0.863
Low	539.38	616.9			616.04	740.87			645	613.24		
Mod	578.38	715.88			554.85	563.17			606.80	600.42		
High	477.62	439.76			558.66	580.26			640.09	617.54		
**Lavender**												
CTR	482.76	328.81	0.421	0.739	861.47	1176.3	0.251	0.832	561.42	608.93	3.171	0.074
Low	471.76	259.85			849.38	1096.8			520.90	521.24		
Mod	515.95	378.09			924.38	1312.9			480.28	582.88		
High	539.52	376.79			882.90	1137.6			931.38	1348.7		

**Jasmine**												
CTR	493.24	406	0.223	0.880	852.28	1075.02	1.859	0.182	664.33	902.18	0.503	0.600
Low	524.43	515.57			712.33	796.97			731.57	1273.8		
Mod	585.48	542.79			617.14	662.72			707.47	887.4		
High	555.29	763.33			663.85	729.31			811.61	1177.4		
**Rose**												
CTR	396.62	345.54	1.668	0.184	660	759.07	3.441	0.022 (0.147)	653.42	690.20	0.930	0.348
Low	441.29	363.38			541.14	616.94			1388.52	4047.2		
Mod	613.14	788.45			616.52	734.83			988.76	1831.1		
High	531.29	514.74			367.34	335.79			665.38	887.9		
**Low-high frequency ratio**												
**Mushroom**												
CTR	6.19	3.98	1.535	0.215	5.77	4.59	1.736	0.169	4.18	2.95	0.145	0.933
Low	6.14	3.87			4.06	3.13			4.52	4.07		
Mod	6.46	5.03			4.83	3.78			4.64	3.43		
High	7.99	5.65			4.38	3.99			4.54	3.61		
**Lavender**												
CTR	5.57	3.89	0.269	0.848	5.35	5.41	1.117	0.338	4.05	3.41	1.029	0.386
Low	4.87	3.97			4.99	4.49			4.74	2.95		
Mod	5.30	2.96			6.01	4.24			4.97	3.90		
High	5.35	2.78			4.36	2.42			4.04	2.48		
**Jasmine**												
CTR	5.94	4.68	0.487	0.693	5.88	7.9	0.948	0.360	4.64	3.65	1.027	0.387
Low	5.80	3.98			4.2	3.69			5.14	4.04		
Mod	5.04	2.79			4.3	3.16			4.46	3.43		
High	5.49	3.16			4.26	3.11			3.99	2.66		
**Rose**												
CTR	7.09	4.57	1.901	0.139 Posthoc-iii:0.008# (Cohen's D - 0.43)	4.47	3.33	1.015	0.377	4.75	5.27	1.141	0.330
Low	6.26	4.21			5.2	4.59			4.50	3.67		
Mod	5.96	3.93			5.6	3.86			4.01	3		
High	5.27	3.82			4.89	4.26			5.61	5.78		

#: While the common practice is not to proceed with post-hoc tests when overall ANOVA result is not significant,several references acknowledge the potential value of this exercise (Hsu JC. Multiple comparisons: theory and methods Chapman & Hall CRC 8 press, Florida,US, 1996, pg177-178). Given that multiple comparisons were a main focus of the present study, it is postulated that they have the power to find differences (CTR:7.09-High:5.27 in this case) between groups even when the overall ANOVA is not significant and such post-hoc results are considered as valid (https://www.graphpad.com/support/faqid/1081/, at 14th Sep 2022). We thus included this particular post-hoc analyses, in the context of above mentioned aspects and the investigatory scope of the present study.

Table 4. Results from the paired comparisons of the baseline (BL), post-stimulation period (Post) looking at all four odours (mushroom, lavender, jasmine and rose) in both cohorts (male, female-follicular, female-luteal) on electrocardiogram (ECG) time-domain (root mean square of successive differences between normal heartbeats - RMSSD and stress index- SI) and frequency-domain (low-frequency power, high-frequency power, low-high frequency ratio) parameters. Cohen – D represented the effect size provided in brackets in the ‘p-value’ column. St.d. = standard deviation, Df. = degrees of freedom, Mean diff. = mean difference between groups, T/Z = t-statistic or Z-score.

	**Male**					**Female-Follicular**					**Female-Luteal**				
	**Mean**	**St.d.**	**Mean diff.**	**T/Z**	**P-value**	**Mean**	**St.d.**	**Mean diff.**	**T/Z**	**P-value**	**Mean**	**St.d.**	**Mean diff.**	**T/Z**	**P-value**
**RMSSD**															
OD1 BL	36.31	13.81	-1.886	-0.74	0.455	40.14	22.87	-9.343	-0.52	0.602	36.34	21.18	-7.753	-0.76	0.444
OD1 Post	38.20	14.57				49.49	43.2				44.09	33.50			
OD2 BL	43.10	19.34	4.624	-1.39	0.164	44.87	36.65	0.824	-0.08	0.931	43.81	35.52	-14.771	-1.11	0.266
OD2 Post	38.48	14.40				44.04	33.01				58.58	61.16			
OD3 BL	43.52	30.51	6.258	-0.33	0.737	44.14	26.98	-7.524	-0.44	0.654	47.99	39.96	1.667	-0.05	0.958
OD3 Post	37.27	15.34				51.67	42.17				46.32	36.05			
OD4 BL	42.37	27.80	6.248	-0.92	0.355	36.02	23.38	-5.538	-0.64	0.520	41.92	39.37	-7.305	-2.01	0.044 (0.17)
OD4 Post	36.12	17.67				41.56	29.46				49.23	45.96			
**Stress index**															
OD1 BL	9.65	2.52	0.69	1.046	0.308	10.73	3.08	0.723	1.04	0.310	12.15	5.47	1.238	1.24	0.228
OD1 Post	8.96	2.96				10.00	3.96				10.91	5.14			
OD2 BL	9.35	3.04	-0.08	0.639	0.639	12	5.42	0.696	-1.30	0.192	11.70	5.11	1.180	1.21	0.239
OD2 Post	9.43	2.75				11.3	5.45				10.52	4.88			
OD3 BL	10.30	4.57	1.03	0.247	0.247	10.17	3.80	0.428	0.66	0.514	10.59	3.73	0.138	0.13	0.894
OD3 Post	9.27	3.25				9.74	3.69				10.45	5.09			
OD4 BL	10.28	4.57	0.49	0.314	0.314	13.05	7.13	1.857	-0.71	0.476	11.07	3.93	0.514	0.68	0.504
OD4 Post	9.79	3.87				11.2	3.84				10.56	4.45			
**Low-frequency power**															
OD1 BL	1945.71	1105.58	-341.33	-1.47	0.140	1484.47	850.28	-784.38	-1.89	0.058	1392.04	1264.14	-497.3	-1.75	0.079
OD1 Post	2287.04	1187.22				2268.85	2094.81				1889.38	1911.80			

OD2 BL	2254.04	1491.73	-373.43	-0.74	0.455	1572.90	1653.08	-901.71	-2.41	0.016 (0.42)	1344.28	1093.68	-682.4	-1.34	0.179
OD2 Post	2627.47	1954.85				2474.61	2462.59				2026.71	2098.17			
OD3 BL	2342.90	2019.42	-145.57	-1.00	0.313	1514.33	1167.60	-354.76	-1.19	0.232	1737.42	1491.33	-625.3	-1.72	0.085
OD3 Post	2488.47	1717.37				1869.09	1033.02				2362.76	1582.64			
OD4 BL	2380.42	2114.90	390.14	-0.07	0.940	1321.90	1272.61	-404.04	-1.68	0.092	2920.66	7425.64	184	-2.13	0.033 (0.03)
OD4 Post	1990.28	1279.01				1725.95	1463.46				2736.66	3298.31			
**High-frequency power**															
OD1 BL	630.19	621.82	97.19	-1.11	0.266	810.04	1184.30	6.904	-1.86	0.063	660.76	685.17	1.523	-1.33	0.181
OD1 Post	533	494.69				803.14	1053.93				659.23	817.94			
OD2 BL	619.80	516.57	13.95	-0.29	0.768	1474.23	2832.47	309.71	-1.00	0.314	760.52	1014.90	-278	-1.23	0.218
OD2 Post	605.85	453.71				1164.52	1596.75				1038.52	1418.23			
OD3 BL	1073.85	2255.65	523.62	-1.06	0.286	837.04	1037.44	111.09	-0.11	0.911	1109.52	1909.65	178.90	-0.33	0.741
OD3 Post	550.23	594.88				725.95	676.09				725.95	676.09			
OD4 BL	781	984.66	237.43	-1.19	0.232	667.90	742.97	71.38	-1.16	0.244	840.38	1463.99	-51.85	-0.46	0.639
OD4 Post	543.57	618.13				596.52	665.88				892.23	1734.80			
**Low-high frequency ratio**															
OD1 BL	4.31	2.65	-1.85	-2.65	0.008 (0.58)	3.67	3.09	-0.736	-1.06	0.289	3.47	2.84	-1.999	-2.41	0.016 (0.55)
OD1 Post	6.16	3.63				4.41	2.09				5.47	4.20			
OD2 BL	4.27	1.95	-0.93	-1.09	0.274	3.07	2.64	-0.906	-1.56	0.117	3.74	2.69	0.353	-0.44	0.654
OD2 Post	5.20	3.37				3.97	2.93				3.38	2.70			
OD3 BL	4.22	3.10	-3.00	-2.76	0.006 (0.79)	4.10	3.73	-0.225	0.00	1	3.02	2.30	-0.808	-2.03	0.042 (0.35)
OD3 Post	7.22	4.38				4.33	3.61				3.83	2.30			
OD4 BL	4.50	3.04	-1.23	-0.82	0.411	3.37	2.60	-1.279	-1.79	0.073	3.85	2.54	-0.830	-1.26	0.205
OD4 Post	5.73	4.47				4.65	3.88				4.68	3.07			

Section 2

Table A1.1 Results from the one-way ANOVA looking at cohort comparisons in the change values from the baseline and post-stimulation periods in an ECG (time-domain: root mean square of successive differences between normal heart-beats - RMSSD and stress index - SI; frequency-domain: low-frequency - LF power, high-frequency - HF power and LF/HF ratio) and eye-tracker (pupil diameter) after the repetitive presentation of **mushroom** odour. St.d. – standard deviation, Df. – degrees of freedom, RMSSD – root mean square of successive differences between normal heartbeats, SI – stress index, LF – low-frequency, HF – high-frequency, Cohort (1 – male, 2 – female-follicular, 3 – female-luteal).

Cohort	Mean	St.d.	Df.	F-statistic	p-value
			**RMSSD**		
1	-1.88	11.82	2, 60	0.356	0.702
2	-9.34	41.76			
3	-7.75	29.10			
			**Stress index**		
1	0.69	3.02	2, 60	0.148	0.863
2	0.72	3.18			
3	1.23	4.55			
			**LF power**		
1	-341.33	1011.47	2, 60	0.594	0.555
2	-784.38	1772.41			
3	-497.33	1090.60			
			**HF power**		
1	97.19	495.33	2, 60	0.138	0.872
2	6.90	913.78			
3	1.52	490.72			
			**LF/HF ratio**		
1	-1.84	3.5	2, 60	0.865	0.426
2	-0.73	3.35			
3	-1.99	3.34			
			**Eye-tracker (pupil diameter)**		
1	-0.08	0.28	2, 60	0.131	0.877
2	-0.11	0.23			
3	-0.11	0.17			

Table A1.2 Results from the one-way ANOVA looking at cohort comparisons in the change values from the baseline and post-stimulation periods in an ECG (time-domain: root mean square of successive differences between normal heart-beats - RMSSD and stress index - SI; frequency-domain: low-frequency - LF power, high-frequency - HF power and LF/HF ratio) and eye-tracker (pupil diameter) after the repetitive presentation of **lavender** odour. St.d. – standard deviation, Df. – degrees of freedom, RMSSD – root mean square of successive differences between normal heartbeats, SI – stress index, LF – low-frequency, HF – high-frequency, Cohort (1 – male, 2 – female-follicular, 3 – female-luteal).

Cohort	Mean	St.d.	Df.	F-statistic	p-value
			**RMSSD**		
1	4.62	12.57	2, 60	1.541	0.223
2	0.82	16.53			

3	-14.77	62.34			
		**Stress index**			
1	-0.07	2.04	2, 60	0.579	0.564
2	0.69	4.43			
3	1.18	4.46			
		**LF power**			
1	-373.42	1619.35	2, 60	0.462	0.632
2	-901.71	1675.20			
3	-682.42	2045.31			
		**HF power**			
1	13.95	423.12	2, 60	1.177	0.315
2	309.71	1807.41			
3	-278	1085.20			
		**LF/HF ratio**			
1	-0.93	3.36	2, 60	1.21	0.305
2	-0.90	3.08			
3	0.35	2.68			
		**Eye-tracker (pupil diameter)**			
1	-0.11	0.33	2, 60	0.032	0.968
2	-0.13	0.21			
3	-0.12	0.20			

Table A1.3 Results from the one-way ANOVA looking at cohort comparisons in the change values from the baseline and post-stimulation periods in ECG (time-domain: root mean square of successive differences between normal heart-beats - RMSSD and stress index - SI; frequency-domain: low-frequency - LF power, high-frequency - HF power and LF/HF ratio) and eye-tracker (pupil diameter) after the repetitive presentation of **jasmine** odour. St.d. – standard deviation, Df. – degrees of freedom, RMSSD - root mean square of successive differences between normal heartbeats, SI – stress index, LF – low-frequency, HF – high-frequency, Cohort (1 – male, 2 – female-follicular, 3 – female-luteal). Posthoc Bonferroni pairwise comparison for significant differences in ANOVA is shown in brackets in the '**p-value**' column.

**Cohort**	**Mean**	**St.d.**	**Df.**	**F-statistic**	**p-value**
		**RMSSD**			
1	6.25	22.88	2, 60	0.929	0.401
2	-7.52	31.31			
3	1.66	42.85			
		**Stress index**			
1	1.03	2.95	2, 60	0.336	0.716
2	0.42	2.95			
3	0.13	4.70			
		**LF power**			
1	-145.57	1332.32	2, 60	0.594	0.555
2	-354.76	1500.36			
3	-625.33	1451.04			
		**HF power**			
1	523.61	2154.73	2, 60	0.437	0.648

2	111.09	1104.04			
3	178.90	1095.20			
		**LF/HF ratio**			
1	-2.99	3.96	2, 60	4.084	0.022 (1 – 2 : p-value = 0.026)
2	-0.22	3.58			
3	-0.80	2.08			
		**Eye-tracker (pupil diameter)**			
1	-0.09	0.22	2, 60	0.469	0.628
2	-0.14	0.19			
3	-0.09	0.21			

Table A1.4 Results from the one-way ANOVA looking at cohort comparisons in the change values from the baseline and post-stimulation periods in an ECG (time-domain: root mean square of successive differences between normal heart-beats - RMSSD and stress index - SI; frequency-domain: low-frequency - LF power, high-frequency - HF power and LF/HF ratio) and eye-tracker (pupil diameter) after the repetitive presentation of **rose** odour. St.d. – standard deviation, Df. – degrees of freedom, RMSSD – root mean square of successive differences between normal heartbeats, SI – stress index, LF – low-frequency, HF – high-frequency, Cohort (1 – male, 2 – female-follicular, 3 – female-luteal).

Cohort	Mean	St.d.	Df.	F-statistic	p-value
		**RMSSD**			
1	6.25	18.71	2, 60	3.154	0.05
2	-5.53	21.32			
3	-7.30	16.74			
		**Stress index**			
1	0.49	2.45	2, 60	0.673	0.514
2	1.85	6.26			
3	0.51	3.46			
		**LF power**			
1	390.143	1686.53	2, 60	0.381	0.685
2	-404.04	1628.98			
3	184	4752.27			
		**HF power**			
1	237.42	684.29	2, 60	1	0.374
2	71.38	648.44			
3	-51.85	662.99			
		**LF/HF ratio**			
1	-1.22	3.98	2, 60	0.106	0.899
2	-1.27	3.39			
3	-0.83	2.91			
		**Eye-tracker (pupil diameter)**			
1	-0.16	0.22	2, 60	0.064	0.938
2	-0.18	0.20			
3	-0.19	0.20			

Table A2.1 Results from the two-way ANOVA looking at cohort (male, females in the follicular and the luteal stage of the menstrual cycle) comparisons in the change values between sham stimulation and each of the three concentrations (low, moderate and high) of each odour in the inter-stimulus washout period in root mean square of successive difference between normal heartbeats - RMSSD scores (time-domain ECG) after acute stimulation. St.d. – standard deviation, Df – degrees of freedom, Cohort (1 – male, 2 – female-follicular, 3 – female-luteal) Conc. – concentration (1 – low, 2 – moderate, 3 – high).

			**Mush**			
**Cohort**	**Conc.**	**Mean**	**St.d.**	**Df**	**F-statistic**	**p-value**
1	1	-2.81	5.97	8, 180	0.692	0.599
	2	-2.89	9.37			
	3	-2.57	5.93			
2	1	0.76	7.32			
	2	-1.16	10.18			
	3	-4.09	12.78			
3	1	0.56	3.75			
	2	2.13	8.77			
	3	-0.14	6.27			
			**Lavender**			
**Cohort**	**Conc.**	**Mean**	**St.d.**	**Df**	**F-statistic**	**p-value**
1	1	-0.54	7.04	8, 180	0.739	0.567
	2	-0.93	9.49			
	3	-0.69	11.65			
2	1	1.95	9.71			
	2	0.99	8.42			
	3	-0.61	12.04			
3	1	1.83	16.83			
	2	4.72	15.24			
	3	-382	18.01			
			**Jasmine**			
**Cohort**	**Conc.**	**Mean**	**St.d.**	**Df**	**F-statistic**	**p-value**
1	1	-0.51	6.57	8, 180	0.318	0.866
	2	-0.81	6.96			
	3	-3.62	15.08			
2	1	1.36	9.80			
	2	1.23	13.37			
	3	0.66	9.30			
3	1	1.62	5.91			
	2	-0.89	4.42			
	3	-3.32	6.02			
			**Rose**			
**Cohort**	**Conc.**	**Mean**	**St.d.**	**Df**	**F-statistic**	**p-value**
1	1	0.22	5.46	8, 180	0.281	0.89
	2	-2.50	7.72			
	3	-1.99	6.05			
2	1	1.04	9.95			
	2	1.98	8.26			
	3	2.84	8.41			
3	1	-7.4	29.36			

	2	-4.21	13.39			
	3	-4.89	19.83			

Table A2.2 Results from the two-way ANOVA looking at cohort (male, females in the follicular and the luteal stage of the menstrual cycle) comparisons in the change values between sham stimulation and each of the three concentrations (low, moderate and high) of each odour in the inter-stimulus washout period in stress index - SI scores (time-domain ECG) after acute stimulation. St.d. – standard deviation, Df – degrees of freedom, Cohort (1 – male, 2 – female-follicular, 3 – female-luteal) Conc. – concentration (1 – low, 2 – moderate, 3 – high).

			**Mush**			
**Cohort**	**Conc.**	**Mean**	**St.d.**	**Df**	**F-statistic**	**p-value**
1	1	0.53	1.65	8, 180	0.213	0.931
	2	0.23	2.08			
	3	0.44	1.94			
2	1	-1.16	6.19			
	2	-0.83	6.01			
	3	-1.06	4.45			
3	1	-1.6	3.79			
	2	-1.11	4.16			
	3	-0.319	3.06			
			**Lavender**			
**Cohort**	**Conc.**	**Mean**	**St.d.**	**Df**	**F-statistic**	**p-value**
1	1	0.47	2.03	8, 180	0.143	0.966
	2	0.22	2.32			
	3	0.29	2.25			
2	1	-2.83	6.28			
	2	-3.16	6.29			
	3	-1.84	5.12			
3	1	0.97	5.78			
	2	0.18	4.26			
	3	1.06	4.18			
			**Jasmine**			
**Cohort**	**Conc.**	**Mean**	**St.d.**	**Df**	**F-statistic**	**p-value**
1	1	-0.30	1.60	8, 180	0.08	0.989
	2	0.14	2.43			
	3	0.16	1.62			
2	1	-0.34	4.40			
	2	-0.11	6.16			
	3	-0.47	5.25			
3	1	-0.07	3.62			
	2	-0.37	5.09			
	3	-0.48	4.00			
			**Rose**			
**Cohort**	**Conc.**	**Mean**	**St.d.**	**Df**	**F-statistic**	**p-value**
1	1	-0.26	1.83	8, 180	0.272	0.896
	2	0.16	2.23			
	3	-0.24	2.70			
2	1	-2.77	7.88			

	2	-3.52	7.29
	3	-2.33	7.04
3	1	0.06	2.22
	2	-1.21	3.40
	3	0.16	3.10

Table A2.3 Results from the two-way ANOVA looking at cohort (male, females in the follicular and the luteal stage of the menstrual cycle) comparisons in the change values between sham stimulation and each of the three concentrations (low, moderate and high) of each odour in the inter-stimulus washout period in low-frequency - LF power scores (frequency-domain ECG) after acute stimulation. St.d. – standard deviation, Df – degrees of freedom, Cohort (1 – male, 2 – female-follicular, 3 – female-luteal) Conc. – concentration (1 – low, 2 – moderate, 3 – high).

**Mush**						
**Cohort**	**Conc.**	**Mean**	**St.d.**	**Df**	**F-statistic**	**p-value**
1	1	-295.38	956.47	8, 180	0.268	0.898
	2	-104.47	1183.78			
	3	-461.14	1258.87			
2	1	228	1172.04			
	2	229.76	1053.66			
	3	249.80	1390.36			
3	1	333.23	1343.73			
	2	48.90	1489.88			
	3	43.42	1581.91			
**Lavender**						
**Cohort**	**Conc.**	**Mean**	**St.d.**	**Df**	**F-statistic**	**p-value**
1	1	116.66	1208.12	8, 180	0.53	0.714
	2	-342	1348.23			
	3	-406.47	1270.25			
2	1	64.52	1472.61			
	2	-238.09	1643.04			
	3	-112.71	1181.67			
3	1	-286.23	831.97			
	2	-94.76	713.28			
	3	-531.14	1153.06			
**Jasmine**						
**Cohort**	**Conc.**	**Mean**	**St.d.**	**Df**	**F-statistic**	**p-value**
1	1	106.71	687.56	8, 180	0.024	0.999
	2	-51.38	656.02			
	3	-185.52	1704.56			
2	1	669.90	2468.36			
	2	654.42	2398.93			
	3	499.38	2008.97			
3	1	111.85	1151.38			
	2	-32.14	814.57			
	3	-255.19	1499.48			
**Rose**						
**Cohort**	**Conc.**	**Mean**	**St.d.**	**Df**	**F-statistic**	**p-value**

1	1	189.76	1506.19	8, 180	0.734	0.57
	2	42.80	1178.94			
	3	-150.38	1774.11			
2	1	241.66	1330.93			
	2	-318.61	790.04			
	3	349.14	1274.42			
3	1	-181.19	1892.29			
	2	-86.47	836.31			
	3	-290.19	1575.45			

Table A2.4 Results from the two-way ANOVA looking at cohort (male, females in the follicular and the luteal stage of the menstrual cycle) comparisons in the change values between sham stimulation and each of the three concentrations (low, moderate and high) of each odour in the inter-stimulus washout period in high-frequency - HF power scores (frequency-domain ECG) after acute stimulation. St.d. – standard deviation, Df – degrees of freedom, Cohort (1 – male, 2 – female-follicular, 3 – female-luteal) Conc. – concentration (1 – low, 2 – moderate, 3 – high).

			**Mush**			
**Cohort**	**Conc.**	**Mean**	**St.d.**	**Df**	**F-statistic**	**p-value**
1	1	-104.85	394.30	8, 180	0.194	0.941
	2	-143.85	481.00			
	3	-43.09	340.33			
2	1	6.381	304.77			
	2	67.57	460.55			
	3	63.76	489.25			
3	1	2.52	269.91			
	2	40.71	423.75			
	3	7.42	343.64			
			**Lavender**			
**Cohort**	**Conc.**	**Mean**	**St.d.**	**Df**	**F-statistic**	**p-value**
1	1	11	249.35	8, 180	1.645	0.165
	2	-33.19	268.44			
	3	-56.76	356.51			
2	1	12.09	541.59			
	2	-62.90	478.09			
	3	-21.42	398.80			
3	1	40.52	363.48			
	2	81.14	474.50			
	3	-369.95	985.75			
			**Jasmine**			
**Cohort**	**Conc.**	**Mean**	**St.d.**	**Df**	**F-statistic**	**p-value**
1	1	-31.19	324.60	8, 180	0.172	0.952
	2	-92.23	364.67			
	3	-62.04	746.54			
2	1	139.95	518.32			
	2	235.14	744.71			
	3	188.42	548.12			
3	1	-67.23	493.79			

	2	-43.14	378.78			
	3	-147.28	640.14			
			**Rose**			
**Cohort**	**Conc.**	**Mean**	**St.d.**	**Df**	**F-statistic**	**p-value**
1	1	-44.66	215.41	8, 180	0.535	0.71
	2	-216.52	512.58			
	3	-134.66	418.21			
2	1	118.85	411.62			
	2	43.47	351.92			
	3	292.65	583.25			
3	1	-735.09	3654.33			
	2	-335.33	1366.90			
	3	-11.95	461.41			

Table A2.5 Results from the two-way ANOVA looking at cohort (male, females in the follicular and the luteal stage of the menstrual cycle) comparisons in the change values between sham stimulation and each of the three concentrations (low, moderate and high) of each odour in the inter-stimulus washout period in low-high frequency ratio - LF/HF ratio scores (frequency-domain ECG) after acute stimulation. St.d. – standard deviation, Df – degrees of freedom, Cohort (1 – male, 2 – female-follicular, 3 – female-luteal) Conc. – concentration (1 – low, 2 – moderate, 3 – high).

			**Mush**			
**Cohort**	**Conc.**	**Mean**	**St.d.**	**Df**	**F-statistic**	**p-value**
1	1	-1.04	6.49	8, 180	0.522	0.72
	2	0.48	4.71			
	3	0.73	3.92			
2	1	1.70	3.79			
	2	0.93	3.29			
	3	1.38	4.87			
3	1	-0.34	3.18			
	2	-0.46	3.90			
	3	-0.36	3.18			
			**Lavender**			
**Cohort**	**Conc.**	**Mean**	**St.d.**	**Df**	**F-statistic**	**p-value**
1	1	3.50	6.45	8, 180	0.086	0.987
	2	2.01	8.66			
	3	3.70	6.07			
2	1	0.36	3.08			
	2	-0.66	5.49			
	3	0.98	4.15			
3	1	-0.68	2.68			
	2	-0.92	3.63			
	3	0.04	2.63			
			**Jasmine**			
**Cohort**	**Conc.**	**Mean**	**St.d.**	**Df**	**F-statistic**	**p-value**
1	1	2.91	9.88	8, 180	0.085	0.987
	2	2.68	5.62			
	3	3.93	8.84			

2	1	1.68	7.81			
	2	1.58	7.26			
	3	1.62	6.85			
3	1	-0.49	2.79			
	2	0.18	3.15			
	3	0.65	2.22			
			**Rose**			
**Cohort**	**Conc.**	**Mean**	**St.d.**	**Df**	**F-statistic**	**p-value**
1	1	-0.63	6.71	8, 180	0.87	0.483
	2	-0.28	8.17			
	3	1.69	7.19			
2	1	-0.72	3.30			
	2	-1.12	2.63			
	3	-0.41	1.54			
3	1	0.25	2.31			
	2	0.74	4.70			
	3	-0.85	4.86			

Table A2.6 Results from the two-way ANOVA looking at cohort (male, females in the follicular and the luteal stage of the menstrual cycle) comparisons in the change values between sham stimulation and each of the three concentrations (low, moderate and high) of each odour in the inter-stimulus washout period in pupil diameter (eye-tracker) after acute stimulation. St.d. – standard deviation, Df – degrees of freedom, Cohort (1 – male, 2 – female-follicular, 3 – female-luteal) Conc. – concentration (1 – low, 2 – moderate, 3 – high).

			**Mush**			
**Cohort**	**Conc.**	**Mean**	**St.d.**	**Df**	**F-statistic**	**p-value**
1	1	-0.023	0.05	8, 180	0.561	0.691
	2	-0.00	0.08			
	3	-0.02	0.04			
2	1	-0.00	0.07			
	2	0.01	0.08			
	3	0.00	0.08			
3	1	-0.02	0.07			
	2	-0.02	0.05			
	3	-0.00	0.05			
			**Lavender**			
**Cohort**	**Conc.**	**Mean**	**St.d.**	**Df**	**F-statistic**	**p-value**
1	1	-0.03	0.12	8, 180	0.452	0.771
	2	-0.01	0.07			
	3	-0.01	0.06			
2	1	0.01	0.06			
	2	0.01	0.06			
	3	0.00	0.04			
3	1	0.02	0.06			
	2	0.01	0.04			
	3	0.01	0.04			
			**Jasmine**			

Cohort	Conc.	Mean	St.d.	Df	F-statistic	p-value
1	1	-0.00	0.07	8, 180	0.249	0.91
	2	0.00	0.10			
	3	-0.01	0.07			
2	1	-0.02	0.05			
	2	0.00	0.06			
	3	0.00	0.07			
3	1	0.00	0.06			
	2	0.00	0.04			
	3	0.00	0.04			

Rose

Cohort	Conc.	Mean	St.d.	Df	F-statistic	p-value
1	1	-0.01	0.05	8, 180	0.685	0.603
	2	0.00	0.07			
	3	-0.04	0.07			
2	1	0.00	0.05			
	2	0.00	0.06			
	3	0.00	0.06			
3	1	0.00	0.06			
	2	0.01	0.07			
	3	-0.01	0.06			

Section 3

Pilot investigation: 1st odour presentation versus baseline

The results for this pilot investigation are presented in **Table A3.1 in the Appendix**. Only specific concentrations of odours showed a significant effect on time-domain ECG parameters of RMSSD and SI. For RMSSD, only a low concentration of *jasmine* showed a significant decrease in the post-odour wash-out period in comparison to the baseline (p-value = 0.008, Cohen's D value of 0.46 indicating a small size). In comparison, all three concentrations of *jasmine* modulated SI scores increasing this parameter in the post-odour wash-out periods in comparison to the baseline period (low concentration - p-value = 0.004, Cohen's D value of 0.69 indicating a medium size; moderate concentration - p-value = 0.008, Cohen's D value of 0.39 indicating a small size; high concentration - p-value = 0.033, Cohen's D value of 0.46 indicating a small size; **Table A3.1 in the Appendix**). Moderate concentration of *lavender* (p-value = 0.003, Cohen's D value of 0.40 indicating a small size) and high concentration of *rose* (p-value = 0.007, Cohen's D value of 0.26 indicating a small size) also showed a significant increase in SI scores in the post-odour wash-out period in comparison to the baseline period. This domain of ECG analysis showed robustness as there were no significant differences present in the sham stimulation post-odour wash-out periods in any of the four odours in both RMSSD and SI parameters. However, the same robustness was not present in the frequency-domain ECG analysis. There was an increased LF/HF ratio towards sympathetic dominance after sham stimulation in comparison to baseline in the *jasmine* group (p-value = 0.029, Cohen's D value of 0.57 indicating a medium size). We also observed a reduced HF power in the low (p-value = 0.038, Cohen's D value of 0.27 indicating a small size) and high (p-value = 0.047, Cohen's D value of 0.36 indicating a small size) concentrations of *lavender* in comparison to baseline and an increased LF/HF ratio towards a sympathetic dominance of the ANS after low concentrations of *mushroom* (p-value = 0.041, Cohen's D value of 0.65 indicating a medium size) and *lavender* (p-value = 0.041, Cohen's D value of 0.71 indicating a medium size) odours (*see* **Table A3.1 in the Appendix**).

Table A3.1 Pilot results from the baseline (BL) compared to the 1st odour presentation washout period for all four concentrations (low, moderate-mod and high concentrations and sham stimulation) of all odours (mushroom, lavender, jasmine, rose) in n = 15 collated in sex (male and female cohorts) and menstrual stage (female-follicular, female-luteal). Paired comparisons (paired t-test or Wilcoxon sign-rank test) were used to compare baseline period with the 1st odour presentation washout periods. Each parameter of ECG time-domain (root mean square of successive differences between normal heartbeats - RMSSD, stress index) and frequency-domain (low-frequency power, high-frequency power and low-high frequency ratio) are reported in separate sections in the table for clarity. St.d. – standard deviation, Z/T – Z-score or t-statistic, degrees of freedom for all data is '14'. Effect size (Cohen's D) calculations are provided in brackets under the 'p-value' column.

Time-domain ECG								
RMSSD								
	Mushroom				Lavender			
	mean	st.d.	Z/T	p-value	mean	st.d.	Z/T	p-value
**BL**	39.87	16.78	1.906	0.077	51.73	37.26	0.738	0.473
**Sham**	35.38	15.98			46.92	22.97		

**BL**	33.96	20.38	1.623	0.127	47.13	37.49	1.785	0.088
**Low**	29.46	15.21			40.74	33.39		
**BL**	33.82	21.59	0.496	0.496	40.68	21.91	0.125	0.125
**Mod**	34.03	19.20			37.08	20.21		
**BL**	43.32	21.32	0.156	0.156	41.51	24.71	0.211	0.211
**High**	39.84	18.38			36.21	20.50		
	**Jasmine**				**Rose**			
**BL**	49.62	43.21	0.478	0.478	34.65	20.88	0.670	0.670
**Sham**	39.20	28.89			30.54	12.49		
**BL**	48.42	22.73	3.455	0.008 (0.46)	44.60	34.13	1.570	0.139
**Low**	38.72	18.46			36.28	23.62		
**BL**	44.85	42.23	0.112	0.112	42.68	21.74	0.139	0.891
**Mod**	36.12	22.94			42.74	20.34		
**BL**	44.70	32.54	0.496	0.496	39.65	44.04	-0.031	0.975
**High**	42	25.61			40.64	51.43		
**Stress Index**								
	**Mushroom**				**Lavender**			
	**mean**	**st.d.**	**Z/T**	**p-value**	**mean**	**st.d.**	**Z/T**	**p-value**
**BL**	10.11	2.62	0.211	0.211	9.38	4.09	0.397	0.397
**Sham**	11.59	4.89			9.74	3.54		
**BL**	12.80	4.99	-1.138	0.274	11.26	5.64	-0.266	0.794
**Low**	14.11	6.17			11.41	5.33		
**BL**	11.02	3.31	-1.882	0.081	10.46	3.12	-3.641	0.003 (0.40)
**Mod**	12.59	4.37			11.86	3.83		
**BL**	9.63	3.55	-0.502	0.624	10.98	3.98	-1.773	0.098
**High**	9.96	3.65			12.49	4.25		
	**Jasmine**				**Rose**			
**BL**	11.44	6.09	-1.063	0.306	11.38	3.53	0.532	0.532
**Sham**	13.30	6.85			11.91	5.01		
**BL**	8.58	2.28	-2.898	0.004 (0.69)	11.68	7.73	-1.307	0.191
**Low**	11.21	4.81			12.78	7.99		
**BL**	10.62	4.36	-3.116	0.008 (0.39)	9.56	5.67	-1.534	0.125
**Mod**	12.65	5.88			10.5	5.34		
**BL**	9.97	3.62	-2.131	0.033 (0.46)	12.54	5.48	-3.123	0.007 (0.26)
**High**	12.62	7.28			14.27	7.30		
**Frequency-domain ECG**								
**Low-frequency power**								
	**Mushroom**				**Lavender**			
	**mean**	**st.d.**	**Z/T**	**p-value**	**mean**	**st.d.**	**Z/T**	**p-value**
**BL**	1528.466	740.08	0.545	0.594	2153.86	1579.48	-0.538	0.599
**Sham**	1384.53	713.87			2437.53	1930.73		
**BL**	1222.33	888.64	-1.578	0.137	1731.60	1633.94	-1.378	0.190
**Low**	1570.80	1263.96			2238.80	2194.19		
**BL**	1423.20	794.15	-0.795	0.427	2019.33	1315.06	0.549	0.592
**Mod**	1410.73	731.90			1873.46	1630.75		
**BL**	2168.13	1584.44	-0.152	0.882	1485.00	971.10	-0.518	0.612
**High**	2213.66	1544.31			1602.40	1256.77		
	**Jasmine**				**Rose**			
**BL**	1999.53	2149.62	-1.305	0.213	1731.33	1779.33	-0.566	0.580

**Sham**	2655.46	3123.61			1850.60	1680.85		
**BL**	1975.40	1247.70	-0.436	0.669	1602.80	1777.12	0.170	0.868
**Low**	2122.26	1323.04			1541.26	1197.73		
**BL**	2005.33	1879.99	1.048	0.312	2167.80	1724.19	-2.034	0.061
**Mod**	1540.86	1061.43			2829.33	1506.20		
**BL**	1822.13	1612.40	-1.500	0.156	3354.73	8776.27	0.798	0.438
**High**	2615.00	3021.62			1686.26	1538.80		
**High-frequency power**								
	**Mushroom**				**Lavender**			
	**mean**	**st.d.**	**Z/T**	**p-value**	**mean**	**st.d.**	**Z/T**	**p-value**
**BL**	512.00	409.72	0.130	0.898	1131.13	1353.06	1.167	0.263
**Sham**	500.53	433.03			987.80	1283.76		
**BL**	490.80	481.81	0.957	0.355	1236.06	1823.13	2.288	0.038
**Low**	384.00	432.90			815.93	1164.50		(0.27)
**BL**	766.66	1357.17	1.090	0.294	1352.00	3012.65	1.196	0.251
**Mod**	528.06	610.51			602.93	732.40		
**BL**	830.20	906.37	1.694	0.112	554.26	488.21	-1.988	0.047
**High**	564.20	567.25			404.26	312.81		(0.36)
	**Jasmine**				**Rose**			
**BL**	1729.20	2939.67	1.337	0.203	574.60	778.71	1.125	0.279
**Sham**	769.33	1090.84			413.93	390.88		
**BL**	931.06	631.70	1.852	0.085	814.73	1104.70	0.786	0.445
**Low**	739.80	797.06			636.86	747.50		
**BL**	1017.66	1977.57	1.052	0.311	738.20	691.63	-1.310	0.211
**Mod**	676.66	892.59			878.86	742.01		
**BL**	1286.26	2616.00	1.234	0.237	825.86	1612.711	1.554	0.143
**High**	716.60	909.64			512.81	967.04		
**Low frequency-highfrequency ratio**								
	**Mushroom**				**Lavender**			
	**mean**	**st.d.**	**Z/T**	**p-value**	**mean**	**st.d.**	**Z/T**	**p-value**
**BL**	4.51	3.83	0.263	0.797	3.61	2.98	-1.783	0.096
**Sham**	4.30	3.08			5.34	4.35		
**BL**	3.32	1.86	-2.251	0.041	3.34	2.27	-2.045-	0.041
**Low**	5.37	4.04		(0.65)	5.68	4.01		(0.71)
**BL**	3.97	2.20	0.377	0.712	3.95	2.47	-0.885	0.391
**Mod**	3.75	2.89			5.00	4.82		
**BL**	4.58	3.58	0.435	0.670	3.81	2.38	-0.511	0.609
**High**	4.20	2.30			4.07	2.33		
	**Jasmine**				**Rose**			
**BL**	4.08	4.29	-2.436	0.029	4.43	2.87	-0.061	0.952
**Sham**	8.32	9.53		(0.57)	4.47	3.62		
**BL**	2.77	2.23	-1.460	0.166	3.42	2.30	-0.754	0.464
**Low**	4.58	4.00			4.34	5.02		
**BL**	3.52	1.82	-0.909	0.363	4.68	3.43	-1.156	0.267
**Mod**	4.88	3.67			6.56	7.46		
**BL**	3.96	4.10	0.174	0.864	3.73	2.04	-1.041	0.316
**High**	3.78	2.80			4.81	4.78		
